# Exported Epoxide Hydrolases Modulate Erythrocyte Vasoactive Lipids during *Plasmodium falciparum* Infection

**DOI:** 10.1128/mBio.01538-16

**Published:** 2016-10-18

**Authors:** Natalie J. Spillman, Varun K. Dalmia, Daniel E. Goldberg

**Affiliations:** a Departments of Medicine and Molecular Microbiology, Washington University School of Medicine, St. Louis, Missouri, USA; b Research School of Biology, The Australian National University, Canberra, Australian Capital Territory, Australia

## Abstract

Erythrocytes are reservoirs of important epoxide-containing lipid signaling molecules, including epoxyeicosatrienoic acids (EETs). EETs function as vasodilators and anti-inflammatory modulators in the bloodstream. Bioactive EETs are hydrolyzed to less active diols (dihydroxyeicosatrienoic acids) by epoxide hydrolases (EHs). The malaria parasite *Plasmodium falciparum* infects host red blood cells (RBCs) and exports hundreds of proteins into the RBC compartment. In this study, we show that two parasite epoxide hydrolases, *P*. *falciparum* epoxide hydrolases 1 (PfEH1) and 2 (PfEH2), both with noncanonical serine nucleophiles, are exported to the periphery of infected RBCs. PfEH1 and PfEH2 were successfully expressed in *Escherichia coli*, and they hydrolyzed physiologically relevant erythrocyte EETs. Mutations in active site residues of PfEH1 ablated the ability of the enzyme to hydrolyze an epoxide substrate. Overexpression of PfEH1 or PfEH2 in parasite-infected RBCs resulted in a significant alteration in the epoxide fatty acids stored in RBC phospholipids. We hypothesize that the parasite disruption of epoxide-containing signaling lipids leads to perturbed vascular function, creating favorable conditions for binding and sequestration of infected RBCs to the microvascular endothelium.

## IMPORTANCE

The malaria parasite exports hundreds of proteins into the erythrocyte compartment. However, for most of these proteins, their physiological function is unknown. In this study, we investigate two “hypothetical” proteins of the α/β-hydrolase fold family that share sequence similarity with epoxide hydrolases (EHs)—enzymes that destroy bioactive epoxides. Altering EH expression in parasite-infected erythrocytes resulted in a significant change in the epoxide fatty acids stored in the host cell. We propose that these EH enzymes may help the parasite to manipulate host blood vessel opening and inflame the vessel walls as they pass through the circulation system. Understanding how the malaria parasite interacts with its host RBCs will aid in our ability to combat this deadly disease.

## INTRODUCTION

Asexual replication of the malaria parasite occurs within the host red blood cell (RBC). After RBC invasion, the parasite exports several hundred proteins beyond the parasite plasma membrane and parasitophorous vacuolar membrane (surrounding the parasite) into the RBC compartment (1, 2). Exported proteins can alter the flexibility of the RBC cytoskeletal network, resulting in increased cell rigidity compared to uninfected RBCs (3). Additionally, several exported proteins are displayed as surface adhesins resulting in cytoadherence of infected erythrocytes to endothelial cell ligands, including intercellular adhesion molecule 1 (ICAM-1) (3, 4). This cytoadherence allows infected RBCs to avoid premature clearance by the spleen, before the 48-h life cycle of the parasite is completed. A pentameric signal sequence, called the *Plasmodium* export element (PEXEL; also known as the host targeting signal) (5, 6), is essential for delivery of most exported proteins. On the basis of identification of this PEXEL sequence, the “exportome” of *Plasmodium falciparum* has been predicted (7–9). Additionally, PEXEL-negative exported proteins have been identified (10), and these proteins add to the number of parasite proteins exported into the RBC. Despite our ability to predict and localize exported proteins, their functions, particularly how they interact/interfere with RBC physiology, remain largely uncharacterized.

RBCs are important regulators of vascular tone. While in circulation, in response to low oxygen content, RBCs release ATP, which interacts with purinergic P2Y receptors on endothelial cells to promote nitric oxide (NO) synthesis (11). Subsequent NO release into the bloodstream inhibits further ATP release from erythrocytes in a negative-feedback loop. The ATP released from erythrocytes also acts in an autocrine manner, activating RBC purinergic P2X_7_ receptors, which in turn activate cytosolic phospholipase A2 (11). RBCs contain significant pools of epoxyeicosatrienoic acids (EETs) (12–15), which are epoxide-containing signaling lipids, released from RBCs in a phospholipase A-dependent manner (16, 17). EETs interact with smooth muscle cells, resulting in activation of Ca^2+^-dependent K^+^ channels, hyperpolarization, and subsequent relaxation/vasodilation (18). EETs (like NO) are anti-inflammatory (19, 20), inhibiting activation of the NF-κB transcription factor in endothelial cells, preventing transcription of endothelium surface receptors, such as ICAM-1 (19).

In healthy individuals, NO and prostacyclins are the main regulators of vasodilation (18); however, in cardiovascular disease states (18), or when NO/prostacyclin biosynthesis is inhibited (21), EETs increase in importance. It is well established that during malaria infection, plasma levels of NO are severely diminished by multiple mechanisms (22–25). The reduction in the bioavailability of NO contributes to the disruption of basal vasoregulation and, thereby, to the cerebral and pulmonary hypertension and reduced blood flow observed in malaria patients (26). When the concentration of NO is reduced, NF-κB-mediated transcription of adhesin ligands is enhanced, promoting infected-erythrocyte sequestration in the microvasculature. Given the decrease in available NO during malaria, we hypothesized that host EET signaling may become more important during malaria infection. If the anti-inflammatory and vasodilatory properties of the EETs could compensate for the decrease in NO, this would be potentially disadvantageous to the parasite (as an “activated” endothelium is beneficial for cytoadherence).

EETs (20-carbon metabolites derived from arachidonic acid), and other anti-inflammatory omega-3 and omega-6 epoxygenated fatty acids, including epoxyoctadecenoic acids (EpOMEs; 18 carbon equivalents derived from linoleic acid) are metabolized by epoxide hydrolases (EHs), converting the active epoxide fatty acids into much less active diols (27, 28). EHs are α/β hydrolases that function both in the detoxification of exogenous epoxides (including toxins/drug metabolites) and in the regulation of lipid signaling epoxides.

*P. falciparum* carries genes that encode four α/β-hydrolases containing PEXEL motifs (5, 8), and all four α/β-hydrolases are present only in the *Laverania* subgenus (*P. falciparum* and *Plasmodium reichenowi*), with no identifiable syntenic orthologues in other *Plasmodium* species (29). In this study, we demonstrate that all four α/β-hydrolases are exported. Two of the α/β-hydrolases share sequence homology with EHs, and our results suggest that, despite having atypical catalytic serine residues, they are capable of hydrolyzing bioactive erythrocyte epoxides to less active 1,2-diols. These enzymes are able to deplete the host erythrocyte of vasoactive epoxides and therefore may play an important role in the vascular biology of malaria infection.

## RESULTS

### Four PEXEL-containing α/β-hydrolases are grouped in two families with different predicted activities.

*P. falciparum* carries genes that encode four α/β-hydrolases containing PEXEL motifs, grouped into two families: α/β-hydrolase group A (PF3D7_0301300 and PF3D7_1401300) and α/β-hydrolase group B (PF3D7_1001400 and PF3D7_1001600) (5, 8). Using a local pairwise alignment algorithm, the two members of α/β-hydrolase family A are 41.4% identical (61.4% similar), and the two members of α/β-hydrolase family B are 53.3% identical (72.5% similar). However, comparisons between family A and B protein sequences reveal less than ~19% identity between the two families.

α/β-Hydrolase fold proteins are a diverse superfamily of enzymes, and despite low sequence similarity at the amino acid level, some distinguishing motifs can aid in the classification of various α/β-hydrolase families. To gain insight into the potential functions of the exported α/β-hydrolases, a PSI-BLAST pseudo-multiple-sequence alignment (comparing the protein of interest to the nonredundant protein data bank) was generated using the online Phyre2 server (30). The top five, unique, non-Apicomplexan hits (with lowest E values) for the *P. falciparum* α/β-hydrolase family A members have the highest similarity to proteins of the epoxide hydrolase family (Interpro IPRO00639; hereafter we refer to PF3D7_0301300 as PfEH1 [*P. falciparum* epoxide hydrolase 1] and PF3D7_1401300 as PfEH2), whereas α/β-hydrolase family B members had the highest similarity to the lysophospholipase/serine aminopeptidase family (Interpro IPRO22742; hereafter we refer to PF3D7_1001400 as PfXL1 [for *P. falciparum* exported lipase 1] and PF3D7_1001600 as PfXL2) (see Table S1 in the supplemental material).

### PfEH1 and PfEH2 are exported from the parasite and are tethered to the erythrocyte cytoskeleton.

**FIG 1:**
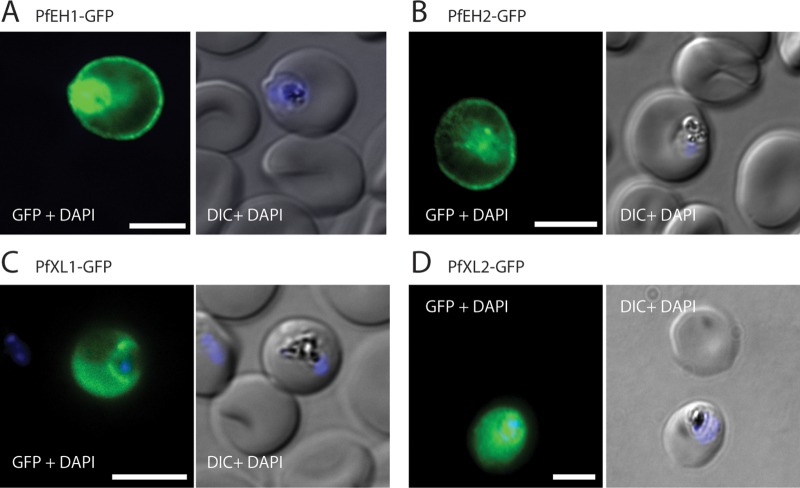
Four α/β-hydrolases are exported into the erythrocyte compartment. Immunofluorescence assay (IFA) of trophozoite/early schizont-stage parasites overexpressing GFP-tagged α/β-hydrolases. Parasites were fixed with paraformaldehyde/glutaraldehyde and stained with anti-GFP (green). The nuclei were stained with 4′,6′-diamidino-2-phenylindole (DAPI) (blue). (A) PfEH1-GFP, (B) PfEH2-GFP, (C) PfXL1-GFP, and (D) PfXL2-GFP. Expression was under control of the constitutive HSP86 promoter (see Fig. S1A and S1B in the supplemental material). The images are representative of at least three independent experiments. Bars = 5 µm. DIC, differential interference contrast.

To investigate the export of the four PEXEL-containing α/β-hydrolases, we generated parasites ectopically expressing a C-terminal green fluorescent protein (GFP) fusion of each α/β-hydrolase (see Fig. S1A and S1B in the supplemental material). For the two α/β-hydrolase family A members, fluorescence was observed at the periphery of the infected erythrocyte and within the parasite (Fig. 1A and B). In contrast, for the two family B members, fluorescence was diffuse throughout the cytosol of the infected RBC (Fig. 1C and D).

**FIG 2:**
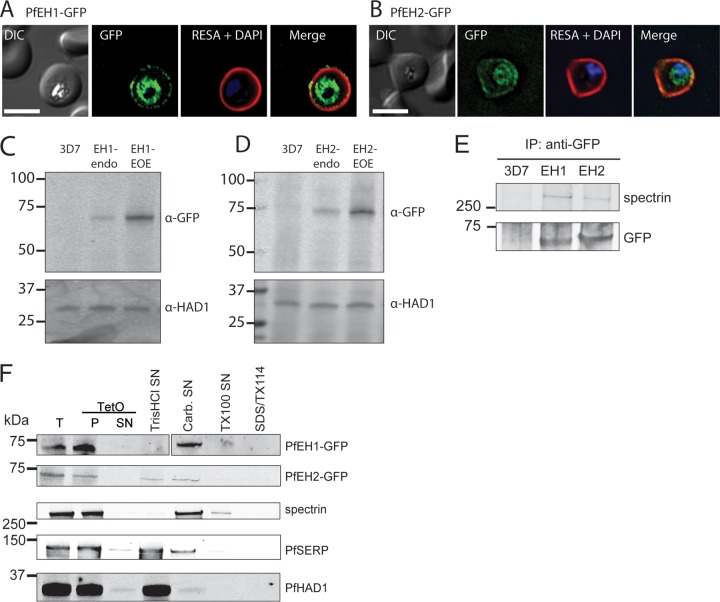
PfEH1 and PfEH2 interact with the erythrocyte cytoskeleton. IFA of paraformaldehyde/glutaraldehyde-fixed, trophozoite/early schizont-stage parasites expressing PfEH1-GFP (A) or PfEH2-GFP (B) under control of the respective endogenous promoter. The GFP-expressing parasites were costained with anti-RESA (red). The nuclei were stained with DAPI (blue). Bars = 5 µm. DIC, differential interference contrast. (C and D) Western blots of PfEH expression in trophozoite-stage parasites. PfEH proteins are not detected in 3D7 parent parasites, barely detected when GFP tagged at the endogenous locus (endo), and highly expressed in the episomal overexpression (EOE) lines. *P*. *falciparum* haloacid dehalogenase 1 (PfHAD1) is used as a loading control. α-GFP, anti-GFP antibody. (E) Immunoprecipitation (IP) of RBCs infected with the 3D7 parent line, PfEH1-GFP or PfEH2-GFP (EOE lines). Complexes were isolated with anti-GFP antibodies, and the final eluate was probed with anti-GFP antibody to confirm pulldown of the PfEH proteins and with human antispectrin antibody. The positions of molecular mass markers (in kilodaltons) are shown to the left of the blots in panels C to E. (F) Sequential fractionation of the 3D7 parent line, PfEH1-GFP, or PfEH2-GFP (EOE lines). The RBC plasma membrane was permeabilized with tetanolysin O (TetO). The total (T) fraction, pellet (P) fraction, and supernatant (SN) fractions are shown in the three leftmost portion of the blots. The pellet fraction was then subjected, sequentially, to hypotonic lysis (Tris HCl) to release soluble parasitophorous vacuole (PV)/parasite/Maurer’s cleft contents, carbonate (Carb.) to release membrane fraction-associated contents, Triton X-100 (TX100) to release membrane contents, and the final pellet was solubilized in Triton X-114 (TX114)/SDS. *P*. *falciparum* serine-rich protein (PfSERP) is a soluble parasitophorous vacuole integrity marker, and PfHAD1 is a soluble parasite integrity marker.

We chose to focus our study on the two putative epoxide hydrolases. We generated parasite integrants expressing the putative EH of interest fused to GFP, under control of the endogenous promoter (see Fig. S1C to S1F in the supplemental material). In asexual parasites, anti-GFP antibody detected PfEH1 and PfEH2 at the periphery of the infected erythrocyte and within the parasite (Fig. 2A and B). The fluorescence at the periphery colocalized with ring-exported surface antigen (RESA) (PF3D7_0102200), an exported protein that interacts with host spectrin at the RBC cytoskeleton (31). Expression from the endogenous locus was low, and by Western blotting, the endogenously tagged PfEH1-GFP or PfEH2-GFP parasites had fusion protein levels that were ~17 ± 5-fold (*n* = 4) and ~6 ± 2-fold (*n* = 4) lower than in the overexpression parasites used in Fig. 1 (Fig. 2C and D).

The PfEH1/2-GFP parasites were used to detect potential interacting partners, which were isolated by pulldown using anti-GFP antibodies and identified using mass spectrometry (see Table S2 in the supplemental material). An enrichment of peptides was observed for α/β spectrin. A similar enrichment for spectrin was not observed in the 3D7 parent, or when PfXL-GFP proteins were used as bait, suggesting that the immunoprecipitation procedure was not nonspecifically pulling down cytoskeletal contents. Chaperones commonly isolated as contaminants (e.g., HSP70) were pulled down equally in all samples, while components of the export machinery (e.g., HSP101) were immunoprecipitated only in the α/β-hydrolase-GFP parasites.

The interaction of the PfEHs with spectrin was confirmed by Western blotting, whereby the GFP-tagged PfEH1/2 were pulled down with anti-GFP antibodies, and the resulting fraction was probed with an antispectrin antibody. Spectrin was present in the immunoprecipitate from GFP-tagged PfEH1/2 lines, but not in the 3D7 parent (Fig. 2E). Consistent with a cytoskeletal interaction, PfEH1 and PfEH2 were both present in the carbonate fraction of a differential solubility assay (Fig. 2F), along with spectrin. In both the immunoprecipitation and fractionation experiments, the size of the GFP-tagged PfEH1/2 protein corresponded to the processed form, after cleavage at the PEXEL motif. The results of the localization and fractionation experiments indicate that PfEH1 and PfEH2 are in close proximity to the erythrocyte cytoskeleton and membrane phospholipids.

**FIG 3:**
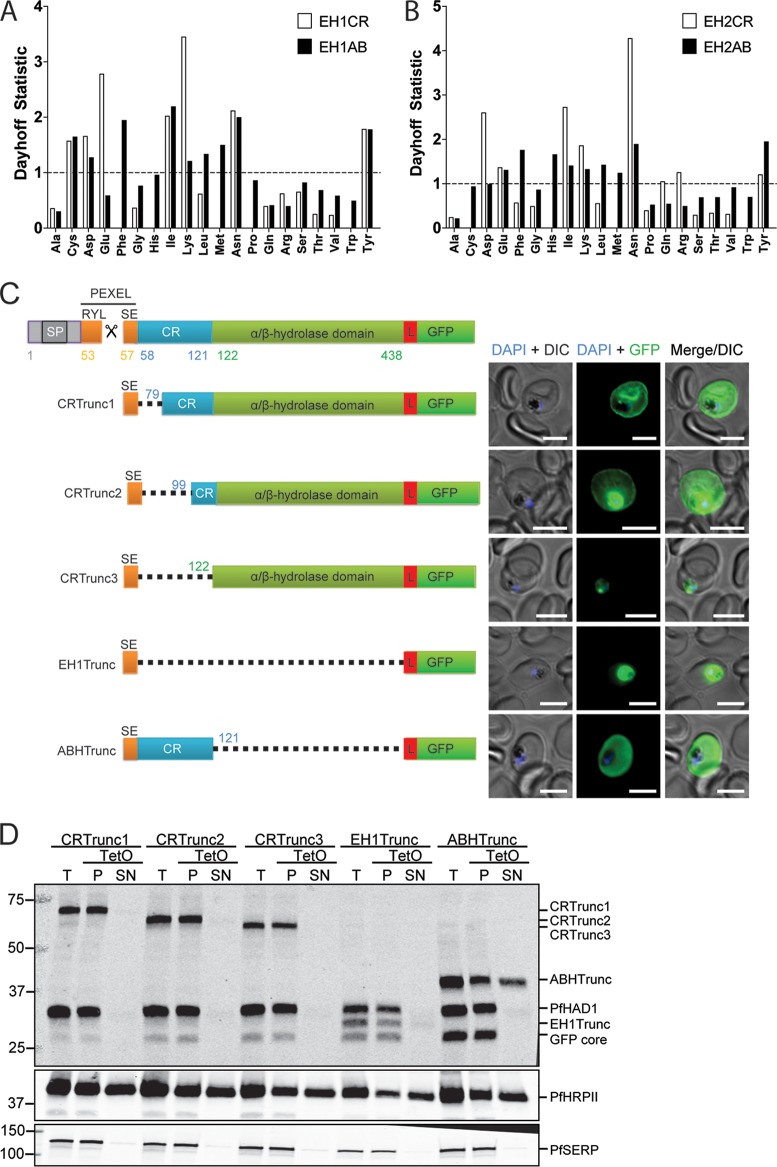
The charged region (CR) of PfEH1 is required for export, and the α/β-hydrolase region is required for cytoskeletal tethering. (A) The amino acid composition of the first 66 residues of mature PfEH1 (EH1CR) was compared to the last 315 residues (EH1AB) using PEPSTATS. The Dayhoff statistic compares the relative occurrence of the amino acid per 1,000 residues to the theoretical Dayhoff statistic for protein composition. A value of 1.0 represents typical frequency. (B) The amino acid composition of the first 49 residues of mature PfEH2 (EH2CR) was compared to the last 333 residues (EH2AB). (C) Schematic representing the PfEH1 truncation (Trunc) constructs. Each construct contains the N-terminal signal sequence and PEXEL motif, as well as a linker region (L) before the reporter GFP. SP, signal peptide. ABH, α/β-hydrolase. (Right) Images from IFA of paraformaldehyde/glutaraldehyde-fixed, trophozoite/early schizont-stage parasites expressing the truncated PfEH1 under control of the HSP86 promoter. The nuclei were stained with DAPI (blue). Bars = 5 µm. DIC, differential interference contrast. (D) Fractionation of the PfEH1-GFP truncation lines with tetanolysin O (TetO). The top blot is probed with both anti-GFP and anti-PfHAD1, a soluble parasite integrity marker. *P*. *falciparum* histidine-rich protein II (PfHRPII) is a soluble exported protein, and PfSERP is a soluble parasitophorous vacuole integrity marker. Lanes: T, total; P, pellet; SN, supernatant. The blots are representative of two or three independent fractionations for each cell line.

Spectrin has previously been identified as a major hub for localization of exported proteins (32), including *P*. *falciparum* erythrocyte membrane protein 1 (PfEMP1) (33), PfEMP3 (34, 35), RESA (31), and knob-associated histidine-rich protein (KAHRP) (36). Spectrin is known to interact with other RBC cytoskeleton members, and also lipids, via charged regions (37–39). We examined the amino acid content of the PfEH proteins and identified a highly charged region in the N terminus of the mature protein dominated by charged Lys and Glu residues in PfEH1 and Asp (and neutral Asn) residues in PfEH2 (Fig. 3A and B). We hypothesized that this charged region (CR) may be important for cytoskeletal tethering, so for PfEH1, this region was truncated to assess the role of the CR in PfEH1 localization (Fig. 3C). Each construct contains the hydrophobic signal peptide and PEXEL sequence fused to the protein domains of interest, followed by a 10-amino-acid linker sequence (PRPGAAHYAA; used previously [40]), and the GFP reporter. When the 64-amino-acid CR was truncated by 22 and 42 amino acids, the reporter was still seen to be exported to the RBC periphery (by both microscopy and tetanolysin O fractionation; Fig. 3C and D), similar to what has been seen with full-length reporter (Fig. 1A and 2D). However, complete truncation of the CR abolished export (Fig. 3C and D), suggesting that the CR contains export-relevant information. Fusing the signal peptide/PEXEL directly to the GFP reporter did not permit export (although as seen previously, the linker sequence may not be sufficient to spatially separate the PEXEL and tightly folded GFP [41–43]). Additionally, when the α/β-hydrolase domain was truncated, the CR region was sufficient to promote export of the reporter construct (Fig. 3C). However, the protein was no longer tethered at the RBC periphery and was found in the tetanolysin O-soluble fraction (Fig. 3D). This indicates that the α/β-hydrolase domain contains information relevant for the tethering of PfEH1 to the cytoskeleton.

### PfEH1 and PfEH2 hydrolyze epoxides, including physiologically relevant signaling lipids.

EHs are distinguished from other α/β-hydrolase fold proteins on the basis of several key motifs (44, 45)—an HGXP motif (part of the oxyanion hole), two tyrosines in the cap domain (functioning in substrate recognition and positioning for the ring-opening reaction), and a GXSmXS/T motif (unknown function) (46). EHs also have a well-studied catalytic triad (nucleophile-acid-base) (44, 45). Amino acid alignments, comparing the sequences of PfEH1/2, PfXL1/2, and the four human EHs, revealed that for PfEH1 and PfEH2, the oxyanion hole motif and GXSmXS/T motif are mostly conserved, and the ring-opening tyrosines (potentially Y316/Y334 in PfEH1 and Y318/Y343 in PfEH2) and catalytic triad are easily identifiable (see Fig. S2 in the supplemental material). A major difference is that PfEH1 and PfEH2 contain a serine (S240 in PfEH1 and S249 in PfEH2) in the position where other EHs contain a catalytic aspartate (44, 45).

**FIG 4:**
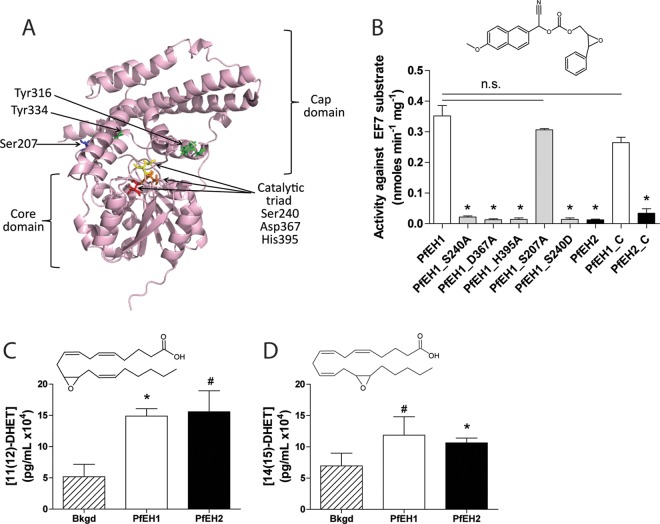
Activity of recombinantly expressed PfEH1 and PfEH2 in *in vitro* epoxide hydrolysis assays and identification of key catalytic residues in PfEH1. (A) Model of PfEH1 (created in Robetta [88], using the structures of *Pseudomonas putida* α/β-hydrolase [PDB accession no. 1ZOI] and human soluble epoxide hydrolase [HsEH2] [PDB accession no. 1S8O]). The variable cap domain and conserved α/β-hydrolase domains are annotated, and key residues are highlighted in contrasting color and shown in stick format. (B) PfEH1, PfEH2, and several PfEH1 mutants were expressed in *E. coli* BL21(DE3). The PfEH proteins were purified by affinity and size exclusion chromatography (two to nine independent inductions/purifications for each condition). Measurement of the fluorescent product (6-methoxy-2-napthaldehyde) generated after hydrolysis of the substrate epoxy fluor 7 (EF7) (structure shown above the graph). All proteins were N-terminally His tagged, except for PfEH1_C and PfEH2_C, which are C-terminally His tagged. Values are means plus SEMs (error bars). Values that are statistically significantly different from the value for PfEH1 (*P* < 0.05 by one-way ANOVA with Tukey’s posttest) are indicated with an asterisk. Values that are not statistically significantly different (n.s.) are also indicated by bars. (C and D) Recombinant PfEH1 and PfEH2 proteins were incubated with 11(12)-EET (structure shown above the graph in panel C) or 14(15)-EET (structure shown above the graph in panel D). Production of the respective diol was measured using ELISA (*n* = 2 to 4, using protein purified from two independent inductions). Values that are statistically significantly different from background (Bkgd) hydrolysis by unpaired *t* test are indicated as follows: *, *P* < 0.05; #, *P* < 0.08. For the 11(12)-EET substrate, the increase in hydrolysis was significant for PfEH1 (*P* = 0.027; *n* = 4) but did not reach significance for PfEH2 (*P* = 0.064; *n* = 4). For the 14(15)-EET substrate, the increase in hydrolysis was significant for PfEH2 (*P* = 0.043; *n* = 3) but did not reach significance for PfEH2 (*P* = 0.077; *n* = 3).

To inform recombinant protein experiments, a model of PfEH1 was generated. The cap domain and core α/β-hydrolase fold domains are identifiable, and the active site residues are clustered near several putative ring-opening tyrosines (Fig. 4A). Robust expression of N-terminal poly(His)-tagged PfEH1 and PfEH2, and of several PfEH1 mutants, was achieved in *E. coli*. The activity of the recombinant enzymes was investigated using the EH reporter substrate epoxy fluor 7 (EF7 [47]). Purified PfEH1 hydrolyzed EF7; however, PfEH2 had very low activity against this substrate (Fig. 4B). PfEH1 activity was significantly diminished when mutations were made in the active site (S240A, D367A, and H395A), but not when a serine distal to the active site (in the cap domain) was mutated (S207A) (Fig. 4B). We attempted to make the PfEH1 active site more closely resemble the active site of human EHs by mutating the catalytic serine to an aspartate (S240D). However, this mutation also significantly reduced the ability of the recombinant enzyme to hydrolyze the EF7 substrate (Fig. 4B). C-terminal poly(His)-tagged PfEH1 and PfEH2 were also expressed and purified, and these enzymes displayed activity that was not significantly different from the activity of the N-terminally tagged proteins.

To examine the ability of the recombinant enzyme to hydrolyze physiological substrates, we incubated the recombinant PfEH1 or PfEH2 with 14(15)-EET or 11(12)-EET and used an enzyme-linked immunosorbent assay (ELISA) to detect formation of the respective diol products 14(15)-dihydroxyeicosatrienoic acid [14(15)DHET] or 11(12)-DHET. Both recombinant PfEH1 and PfEH2 enzymes increased the formation of the diol product above levels generated by spontaneous hydrolysis (Fig. 4C and D).

### PfEH1 and PfEH2 can be disrupted *in vitro*.

**FIG 5:**
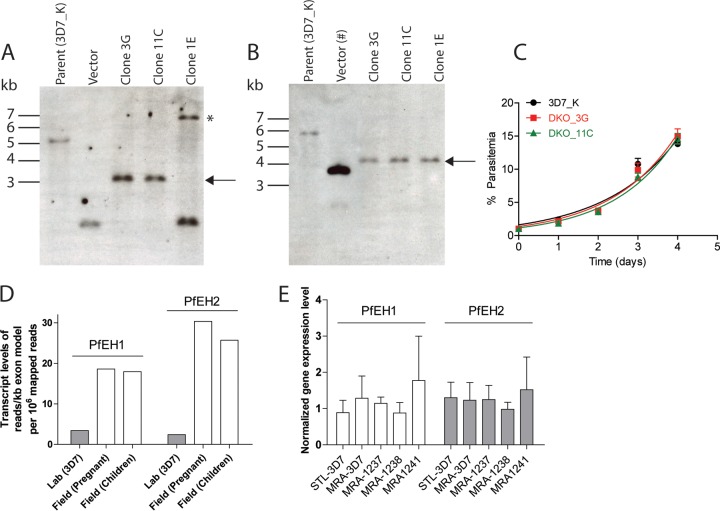
PfEH1 and PfEH2 can be knocked out in cultured parasites. (A and B) Southern blots of digested genomic DNA from the parent 3D7 line, vector, and double-knockout clones. Analysis at both the PfEH1 (A) and PfEH2 (B) loci demonstrate the simultaneous disruption of both genes in clones 3G and 11C, but not in clone 1E. The black arrows to the right of the blots indicate the expected size for correct integration, and the asterisk indicates a band of unknown origin. In panel B, the pUF-TK_PfEH2 vector [Vector (#)] was used instead of pCC1_PfEH2 (called pABH-DKO in reference 89). The schematic representations of the integration events are outlined in Fig. S2 in the supplemental material. (C) Growth of the 3D7 parent line and the two double-knockout (DKO) clones over 4 days. No differences were observed in growth between these three cell lines, with this graph representative of data from three independent experiments. The doubling times are 1.14 ± 0.12 days for the 3D7 parent, 1.16 ± 0.10 days for double-knockout clone 3G, and 1.26 ± 0.12 days for double-knockout clone 11C (not significantly different [*P* > 0.53] using pairwise *t* tests; *n* = 3 for each). The smooth curve is the fitted exponential growth equation. (D) Transcript levels of PfEH1 and PfEH2 in unsynchronized 3D7 laboratory strain compared to field isolates from malaria-infected pregnant women or malaria-infected children. The data shown were taken from Vignali et al. (48). (E) Normalized expression of PfEH1 or PfEH2 in two laboratory strains (Saint Louis STL-3D7 or MRA-3D7) and three laboratory-adapted field isolates from Cambodia (MRA-1237, MRA-1238, and MRA-1241). Quantitative RT-PCR was performed using probes against PfEH1 and PfEH2, with actin as a reference gene. Values are normalized to the average expression in STL-3D7 and MRA-3D7. Data were calculated from three independent RNA preparations (means plus SEMs [error bars]) and are not significantly different using a two-way ANOVA (*P* > 0.05).

To assess the *in vitro* importance of PfEH1 and PfEH2, parasite lines were generated in which either individual locus was disrupted by double-crossover recombination, with insertion of the yeast dihydroorotate dehydrogenase (yDHODH) selection cassette (strategy in Fig. S3A to S3D in the supplemental material). Neither single-knockout parasite line displayed any obvious morphological changes or significant alteration of growth rate compared to the 3D7 parental line (Fig. S3E). A double-knockout line was created by knocking out PfEH1 and -2 sequentially using orthogonal drug selection cassettes. Southern blotting confirmed the successful generation of PfEH1/2 double-knockout clones (Fig. 5A and B). As was the case for the single knockouts, the double knockout also had no observable morphological changes or alteration in growth rate (Fig. 5C). PfEH1 and -2 do not appear to be required for growth in culture.

Previously reported expression profiling revealed that field isolates (with minimal *in vitro* culturing) have increased expression levels of PfEH1 and PfEH2 genes (5- to 10-fold; data from genome-wide RNA analysis in Vignali et al. [48]) (Fig. 5D). This suggested that expression of PfEH1/2 may be beneficial *in vivo*. We examined three field isolates that had been adapted to *in vitro* culture through cryopreservation/thawing three times before analysis (49, 50). After a limited number of additional cryopreservation/thawing steps in our laboratory, we isolated RNA from these parasites and examined the relative expression of PfEH1 and PfEH2 in these culture-adapted field isolates compared to two 3D7 laboratory lines. Quantitative reverse transcription-PCR (RT-PCR) demonstrated that there was no significant difference in expression of either PfEH gene in these field lines adapted to *in vitro* culture (Fig. 5E). These results suggest that the ectopic overexpression constructs of PfEH1/2 (Fig. 1A and B) may be more representative of *in vivo* expression levels (i.e., high expression), whereas laboratory-adapted lines have reduced requirement to express PfEH1/2 (i.e., low expression).

### Overexpression of PfEH1 or PfEH2 results in a reduction in erythrocyte epoxide signaling lipids.

**FIG 6:**
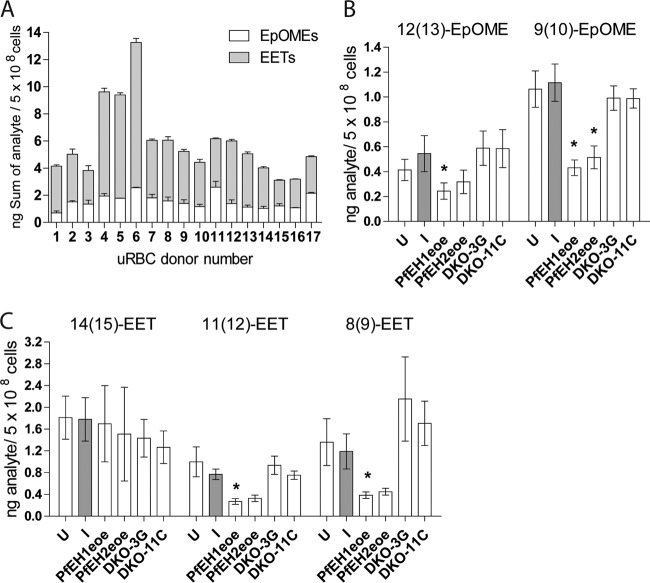
LC/MS/MS measurement of epoxide-containing fatty acids in phospholipid preparations from parasitized RBCs. Internal standards 5(6)-EET-d_11_, 8(9)-EET-d_11_, 11(12)-EET-d_11_, 14(15)-EET-d_11_, and 9(10)-EpOME-d_4_ were added to each sample, phospholipids were extracted with 2:1 CHCl_3_−CH_3_OH plus 0.1 mM triphenylphosphine, and fatty acids were released using phospholipase A2. Four-point calibration curves were generated using the deuterated standards to quantify the epoxide analytes. (A) Sum of 8(9)-EET, 11(12)-EET, 14(15)-EET, 9(10)-EpOME, and 12(13)-EpOME in uninfected RBCs (uRBC). Samples were run twice, and the error bars depict range/2. (B and C) 9(10)-EpOME and 12(13)-EpOME (B) and 8(9)-EET, 11(12)-EET, and 14(15)-EET (C) concentrations in phospholipids of uninfected RBCs (U) and RBCs infected with 3D7 parent (I), PfEH1-GFP (PfEH1eoe), PfEH2-GFP (PfEH2eoe), and PfEH1 PfEH2 double-knockout (DKO) clones 3G and 11C. The values shown are calculated from 3 to 9 independent phospholipid preparations per condition (mean ± SEM). Values that are statistically significantly different from the value for infected RBCs (*P* < 0.05 by paired *t* tests) are indicated by an asterisk. For PfEH1, there was a significant reduction in 12(13)-EpOME (*P* = 0.049; *n* = 4), 9(10)-EpOME (*P* = 0.043; *n* = 4), 11(12)-EET (*P* = 0.0154; *n* = 4), and 8(9)-EET (*P* = 0.034; *n* = 4), while for PfEH2, several compounds showed reductions, but the decrease reached statistical significance only for the 9(10)-EpOME analyte (*P* = 0.028; *n* = 4).

To explore the effect of altering PfEH1 or PfEH2 expression on the composition of erythrocyte epoxide signaling lipids, fatty acid preparations were isolated from the phospholipid fraction of erythrocytes infected with the PfEH1/2 double-knockout clones (from Fig. 5) or the PfEH1- or PfEH2-overexpressing lines (from Fig. 1). These lipid preparations were analyzed using a targeted liquid chromatography coupled to tandem mass spectrometry (LC/MS/MS) approach to measure three EET regioisomers [14(15)-EET, 11(12)-EET, and 8(9)-EET] and two EpOME regioisomers [12(13)-EpOME and 9(10)-EpOME]. It has been previously reported that the concentration of EETs in erythrocytes varies significantly between individual blood donors (by 2- to 3-fold) (14). We also observed a 2- to 3-fold variation in RBC epoxy fatty acid content between blood donors used over the course of this study (Fig. 6A). Therefore, it was essential to pair all analyses and use blood from the same donor/donor pool for culture of a parasite line within an experiment. When RBCs were infected with the 3D7 parasite line, there were no significant differences in any of the five analytes measured compared to the control uninfected RBCs (Fig. 6B and C). There were also no significant differences in epoxy fatty acid content comparing infection with the 3D7 parent to infection with the PfEH1/2 double-knockout clones (Fig. 6B and C). However, in the PfEH1- or PfEH2-overexpressing lines, compared to the 3D7 parent line, there was a substantial reduction of several epoxy signaling lipids. These results are consistent with PfEH1 and PfEH2 altering the concentration of epoxy signaling lipids within the infected erythrocytes.

## DISCUSSION

The physiological functions of the several hundred *P. falciparum* proteins exported into the host cell remain poorly characterized. In this study, we show that four α/β-hydrolases are exported into the RBC and that two of these, PfEH1 and PfEH2, have epoxide hydrolase activity and are capable of hydrolyzing bioactive RBC lipid epoxides to less active diols. We propose that the malaria parasite can reduce lipid epoxide signaling of the host RBC, a disruption that may lead to perturbed vascular signaling and inflammation.

In circulating RBCs, stimuli, such as shear stress or stimulation of the purinergic P2X_7_ receptors by ATP, activate phospholipase A_2_, which releases EETs from their esterified form in membrane phospholipids into a soluble free fatty acid form in the cytosol (16, 17). Following de-esterification, the soluble EETs are then released from the RBCs in a process requiring the cystic fibrosis transmembrane regulator (CFTR), pannexin-1, and the voltage-dependent anion channel (VDAC) (17, 51). Infection with *P. falciparum* is known to activate several RBC ATP release pathways, including VDAC (52–54). Therefore, it is possible that EET de-esterification and release is activated during *P. falciparum* infection. The observed localization of PfEH1 and PfEH2 at the periphery of the infected RBC would facilitate the rapid metabolism of released epoxy fatty acids.

Aside from proximity to substrate, the functional significance of the observed interaction of the PfEHs with the RBC cytoskeleton is unknown. Further work is necessary to demonstrate a direct interaction between the PfEHs and spectrin (or another cytoskeletal component) and to investigate whether the PfEHs interact with other malarial proteins at the cytoskeleton. Our analyses of truncation mutants of PfEH1 suggested that the α/β-hydrolase domain, and not the highly charged mature N-terminal region, is essential for the cytoskeletal interaction. Other exported proteins, where there is direct evidence for their interaction with the cytoskeleton, also contain charged regions or charged repeats, including KAHRP (32, 33, 36) and lysine-rich membrane-associated PHISTb protein (LYMP) (55, 56); however, the function of their charged regions is unstudied. The highly charged N-terminal region in PfEH1 was necessary to facilitate export. Three different charged PfEH1 mature N termini (full-length/ABHTrunc, CRTrunc1, and CRTrunc2) permitted export into the RBC, but the two truncations without the charged N termini (CRTrunc3 and EH1Trunc) did not. Charge differences between the N and C termini of exported proteins were previously proposed to contribute to the export process (57, 58); however, it was subsequently shown that scrambling the N-terminal charged regions reduces export (42), suggesting that the sequence was also a driving factor. It is also possible that for the two truncations that were not exported, there was a steric block along the secretory pathway. The requirement to separate the PEXEL motif from the tightly folded GFP core, in a sequence-independent manner, has been described (41), with the hypothesis being that if the N-terminal signal is too short, it cannot be efficiently recognized and/or unfolded by chaperones/export machinery in the parasitophorous vacuole. It is possible that the export signal is too tightly associated with GFP (for EH1Trunc) or with the structured α/β-hydrolase domain (for CRTrunc3) to be recognized for successful export. Further dissection of the PfEH1 (and PfEH2) protein is necessary to clarify the requirements for export and the requirements for cytoskeletal interaction in more detail.

In this study, we demonstrate that PfEHs were able to hydrolyze epoxides in *in vitro* experiments with recombinantly expressed PfEHs and also in parasite-infected RBCs. In the assays reported here, PfEH1 and PfEH2 behaved similarly (the exception being that PfEH2 did not hydrolyze the EF7 fluorescent reporter substrate, though EHs with poor activity against nonphysiological fluorescent reporters but higher activity against endogenous substrates have been reported [59]). If the localization and ability to hydrolyze EETs/EpOMEs are similar, then it is unclear why *P. falciparum* would express two EHs. One possibility is that the two enzymes are not redundant, as they have different substrate selectivities. Alternatively, they may be important at different stages of the intraerythrocytic life cycle. Consistent with this hypothesis, previous studies indicated that PfEH2 may be upregulated in the gametocyte (sexual) stage (5, 60). PfEHs may also be important in the parasite mosquito stages as EETs and EpOMEs are taken up in the mosquito blood meal. Mosquito EHs can reduce EET/EpOME levels (61, 62), and modulating EH activity alters the mosquito immune response, which may alter parasite viability (63). Both PfEH1 and PfEH2 are upregulated in field isolates (48) (Fig. 5D); therefore, the PfEH-overexpressing lines created in this study may be more physiologically relevant to study EH function compared to laboratory-adapted lines, such as 3D7, which expresses very low levels of EH enzymes and behaved like our PfEH1 double-knockout line.

One challenge in studying epoxide-containing signaling lipids is the instability of these metabolites. The level of epoxy fatty acids in our cultured RBCs (~10 ng EETs/10^9^ RBCs) was lower than previously reported for “fresh” RBCs (~33 ng EETs/10^9^ RBCs) (14). Goulitquer and colleagues reported EET levels to be quite unstable, with a rapid loss of approximately 20 to 30% in the first hour after the blood draw, though the decrease is slower at 4°C (14). Our parasites are cultured at 37°C for several days, likely leading to the decreased level of the epoxy fatty acids reported here. Examination of epoxy fatty acid profiles in freshly drawn blood from healthy individuals compared to patients infected with *P. falciparum* would provide further evidence of the significance of PfEHs in altering RBC physiology. A previous report examined the vasoactivity of RBCs from *P. falciparum* malaria-infected patients in Benin and found increased contractile responses in the malaria-infected RBCs compared to uninfected samples (64). This result is consistent with a decrease in vasodilatory signals (e.g., EETs) upon malaria infection. Interestingly, the four α/β-hydrolases are part of a “greatly expanded exportome” found in *P. falciparum* (and *P. reichenowi*) (8). These species-specific exported proteins are thought to be involved in the trafficking and display of the virulence ligand PfEMP1 (8). It is possible that the PfEHs contribute to the vascular inflammation and display of PfEMP1-compatible adhesins, although this hypothesis requires experimental investigation.

*P. falciparum* is not the only pathogen to use an EH to alter host function. EH activity has been extensively studied in *Pseudomonas aeruginosa*, a bacterium that causes opportunistic pulmonary infections, particularly in some cystic fibrosis cases. *P. aeruginosa* secretes the “CFTR inhibitory factor,” or Cif EH (65), and the Cif EH enters into the airway epithelial cells. Although Cif has classical EH activity (66, 67), it also inhibits CFTR endocytic recycling (66, 68), leading to a decrease in apical CFTR levels, exacerbating cystic fibrosis symptoms. The Cif EH has a noncanonical active site, including having a glycine instead of a proline in the HGxP motif (69). Differences in reaction mechanism (compared to the well-studied human soluble EH [sEH]) may be necessary to facilitate the non-EH Cif activities. The PfEHs also have a noncanonical active site, as they lack the aspartate nucleophile found in characterized EHs. It has previously been hypothesized that an EH using a serine nucleophile would result in an “overly stable reaction intermediate” (70); however, the nucleophile alone is not the sole residue driving EH activity (71). Solving the crystal structure of PfEH1 or PfEH2 would allow an examination of the active site features. The full range of PfEH substrates and functions requires further characterization.

It is possible that the PfEHs, like Cif, may have other non-EH activities and that these activities may require a serine nucleophile (a residue facilitating an incredibly diverse array of at least 17 α/β-hydrolase enzymatic activities [72]). Consistent with this, it was recently described that PfEH2 also possesses an S33 class proline aminopeptidase activity (73). The peptidase activity of PfEH2 is low (reported *K_m_* of 403 µM and *k*_cat_/*K_m_* of ~28 M^−1^ s^−1^ [73]) compared to other characterized proline aminopeptidases (74, 75). The physiological substrate(s) and relevance of this activity are yet to be determined. Epoxide hydrolases are common nonpeptidase homologues of the S33 class peptidases (76, 77), due to the broad hidden Markov matrices used to identify peptidase members (78). Functional promiscuity in the α/β-hydrolase family has been reported (79), and investigation into whether the PfEHs possess multiple activities requires further study. Interestingly, in the study on the proline aminopeptidase of PfEH2, the infected RBC (iRBC) membrane of the PfEH2 knockout parasites was more elastic than that of the parent parasites (73), which may reflect a change in the phospholipid composition of these parasites.

Pharmacological inhibition of PfEHs *in vivo* merits further investigation. Promoting a “quiescent” endothelium and normal vasoreactivity would be disadvantageous to the parasite, as fewer endothelial adhesins displayed would result in less cytoadherence and increased splenic clearance of parasitized RBCs. Multiple EH inhibitors are in development (67, 80, 81), with several currently in clinical trials (ClinicalTrials.gov identifier [ID] NCT00847899, NCT01762774, and NCT02006537). If epoxy signaling lipids are important for reducing *P. falciparum* virulence *in vivo*, there is also a possibility that these inhibitors may be repurposed as antimalarials. Aside from modulating the host EH enzymes, it may also be possible to directly target the PfEHs, and work in this area is ongoing. Understanding the regulation of RBC epoxide-containing signaling lipids by parasite EHs will pave the way for the identification and validation of new drug targets within this pathway.

## MATERIALS AND METHODS

See supplemental material for full details.

### *P. falciparum* culture.

*P. falciparum* line 3D7 was cultured at 2% hematocrit in O^+^ erythrocytes in RPMI 1640 supplemented with 0.5% (wt/vol) Albumax II (40). Cultures were grown statically in 5% O_2_, 5% CO_2_, and 90% N_2_. As required, cultures were synchronized with 5% (wt/vol) sorbitol (82). Parasites generated in this study include C-terminal GFP-tagged PfEH1 or PfEH2 (expression under control of the endogenous or heat shock protein 86 [HSP86] promoters; see Fig. S1 in the supplemental material), C-terminal GFP-tagged PfXL1 or PfXL2 (under control of the HSP86 promoter; Fig. S1), and single- or double-knockout parasites of PfEH1 and PfEH2 (Fig. S3). Infected erythrocytes (mid-stage trophozoites) were enriched to >95% parasitemia using LD columns on a QuadroMACS separator.

### Immunofluorescence assay, cell fractionation, immunoprecipitation, mass spectrometry, and Western blotting.

Indirect immunofluorescence assay (IFA) (83, 84) and sequential permeabilization of enriched, infected erythrocyte compartments (42) was performed as previously described. For immunoprecipitation, parasite lysates were incubated with protein A Dynabeads (Molecular Probes) and nutated with rabbit polyclonal anti-GFP (ab6556; Abcam) (2 to 5 µg) for 16 h (4°C).

### Epoxide hydrolase activity assays.

Epoxy fluor 7 (EF7), EETs, and EpOMEs were from Cayman Chemicals. Fluorescence/absorbance was monitored using an EnVision Multilabel plate reader (PerkinElmer). EH activity was measured using the EF7 substrate (47) in a 200-µl reaction mixture containing 5 µM EF7 and 10-µg recombinant enzyme in phosphate-buffered saline (PBS) (expressed in *E. coli* as previously described [85]). Diol formation (measuring 6-methoxy-2-naphthaldehyde production) was quantified by measuring fluorescence (excitation at 340 nm and emission at 450 nm). Calculation of the initial (linear) rates of substrate hydrolysis was determined using GraphPad Prism software (v5). EH activity against the 11(12)-EET and 14(15)-EET regioisomers was measured using the 11(12)- or 14(15)-DHET ELISA kit, according to the manufacturer’s protocol (Detroit R&D), with some modifications. Reaction mixtures (100 µl) contained 6 or 12 µM EET and 100 µg EH recombinant enzyme in PBS and were incubated at 37°C for 1.5 h. Sample buffer (900 µl) was added to the reaction mixture, and 100 µl was loaded onto the ELISA wells in duplicate. Absorbance was measured at 450 nm.

### Gene expression by quantitative reverse transcription-PCR (qRT-PCR).

Strains used were *P. falciparum* (“Saint Louis” 3D7 [86] or “MRA-102” 3D7 [obtained through BEI Resources Repository, NIAID, NIH: contributed by D. J. Carucci/A. Craig]) and Cambodian field isolates (49, 50) (MRA-1237, -1238, and -1241 [strains IPC 3663, 4884, and 4912, respectively, obtained through BEI Resources Repository, NIAID, NIH: contributed by D. Menard]). Tightly synchronized parasites were saponin isolated (0.05% saponin in PBS; 5 min, room temperature) and RNA was isolated using the RNeasy minikit (Qiagen) with on-column DNase treatment (15 min, room temperature). One-step RT-PCR was performed using the TaqMan fast virus one-step RT-PCR master mix (Life Technologies) using an Applied Biosystems 7500 Fast real-time PCR system. Primer sets and probes were designed (IDT PrimeTime quantitative PCR [qPCR] assay) for PfEH1, PfEH2, and actin (PF3D7_1246200) (see Table S1 in the supplemental material). The relative expression levels of PfEH1 and PfEH2 were calculated using the comparative threshold cycle (*C_T_*) method (87) using actin as a reference control gene. Normalized gene expression was calculated as 2^−ΔΔ*CT*^ where Δ*C_T_* = *C_T_* (actin) − *C_T_* (PfEH) where *C_T_* (actin) is the threshold cycle of actin and ΔΔ*C_T_* = Δ*C_T_* (average STL/MRA 3D7) − Δ*C_T_* (Cambodian isolate).

### Analysis of EET and EpOME metabolites by LC/MS/MS.

Lipid mass spectrometry was performed at the Metabolomics Facility at Washington University (P30 DK020579). Enriched (>95% parasitemia, QuadroMACS), trophozoite-stage infected RBCs (5 × 10^8^ cells) were washed three times in PBS, resuspended in 200-µl water, and internal standards were added [5 ng each of 5(6)-EET-d_11_, 8(9)-EET-d_11_, 11(12)-EET-d_11_, 14(15)-EET-d_11_, and 9(10)-EpOME-d_4_]. Phospholipids were extracted twice using 2:1 CHCl_3_-CH_3_OH with 0.1 mM triphenylphosphine, hydrolyzed using phospholipase A2, and extracted twice with ethyl acetate as described previously (13). The dried extract was stored at −80°C until analysis, when it was suspended in 100 µl of methanol. After removing particulates, 10 µl of the supernatant were injected onto an online trapping liquid chromatography coupled to tandem mass spectrometry (LC/MS/MS) system. This system consists of a Leap PAL autosampler, four Shimadzu high-performance liquid chromatography (HPLC) 20AD pumps, and an API-4000 quadrupole mass spectrometer. Two types of Thermo-Keystone C_18_ betasil trapping column (2 by 10 mm; 5 µm) and C_18_ betasil analytical column (2 by 100 mm; 3 µm) were used for the analyses. The mobile phases were 1% formic acid in water and acetonitrile for the trapping column and 10 mM ammonium formate in water and acetonitrile for the analytical column. Solvent gradient programs were used for trapping and analytical runs. Negative ion multiple reaction monitoring (MRM) mode was used to detect all analyte signals. For quantification of all analytes, four point calibration standards of all analytes were prepared in the presence of the deuterated internal standards and were injected onto the system for calibration curves. Analyst 1.5.2 version software was used for operation and quantification.

### Statistical analyses.

Data are expressed as means ± standard errors of the means (SEMs). Statistical comparisons were made using a *t* test (paired/unpaired as indicated), one-way analysis of variance (ANOVA) with Tukey’s posttest. Statistical analyses were performed in GraphPad Prism v 5.01.

## SUPPLEMENTAL MATERIAL

Figure S1  Generation of the GFP-tagged lines. (A) Schematic representation of the pEOE construct used to overexpress the protein of interest. The vector contains the human dihydrofolate reductase (hDHFR) positive-selection cassette, with selected parasites expressing the gene of interest under the strong, constitutive hsp86 promoter. (B) Schematic representation of the pTEOE construct used to overexpress the protein of interest via transposase-mediated integration. The vector contains the *piggyBac* element containing inverted terminal repeats (ITR) (90) and the hDHFR positive-selection cassette. The pHTH vector contains a cassette for transient expression of the *piggyBac* transposase (90), facilitating genomic integration of the pTEOE sequence, leading to stable expression of the gene of interest under the strong, constitutive hsp86 promoter. (C and E) Schema outlining the strategy for replacement of the 3′ region of the gene of interest by single-crossover homologous recombination. The pPM2GT vector (40) was used to append GFP to the 3′ end of the gene of interest, with positive selection mediated by the hDHFR selection cassette. The restriction digestion sites and the resulting expected sizes of DNA fragments that were utilized to characterize the locus by Southern blot analysis are labeled, with the probe represented by an orange bar. (D and F) Southern blots of digested genomic DNA from the 3D7 parent parasite line, vector, and tagged clones, showing correct integration of the GFP tag. Arrows indicate the expected size for correct integration, and asterisks indicate bands of unknown origin. In both PfEH1- and PfEH2-GFP lines, the plasmid band remains present, indicating integration of a concatemerized plasmid. Download Figure S1, JPG file, 0.9 MB

Figure S2  Amino acid alignment of PfEH1 and PfEH2 compared to known EH enzymes. Clustal Omega alignment of PfEH1, PfEH2, PfXL1, and PfXL2 with the equivalent regions in the four human EHs (*Homo sapiens* EHs [HsEHs]) (HsEH1, GenBank accession no. NP_001970.2; HsEH2, GenBank accession no. AAC41694.1; HsEH3,GenBank accession no. AAI15003.1; HsEH4, GenBank accession no.NP_775838.3). HsEH1 is the microsomal EH (HsEHm), and HsEH2 is the soluble EH (HsEhs). Identical residues are colored black, and functionally conserved residues are colored gray. The regions aligned represent the putative (i) oxyanion hole motif, (ii) GxGxS/T motif of unknown function, (iii) catalytic nucleophile, (iv) catalytic acid residue, and (v) catalytic base residue. Download Figure S2, JPG file, 1.1 MB

Figure S3  Generation of PfEH1 and PfEH2 single-knockout lines. (A, C, and F) Schema outlining the strategy for disruption of the gene of interest by double-crossover homologous recombination. The pUF-TK vector contains the yDHODH cassette for positive selection and the herpes simplex virus thymidine kinase (TK) cassette for negative selection. The pCC-1 vector contains the hDHFR cassette for positive selection and the yeast cytosine deaminase and uridyl phosphoribosyl transferase cassette for negative selection. The restriction digestion sites and the resulting expected sizes of DNA fragments that were utilized to characterize the locus by Southern blot analysis are labeled, with the probe represented by an orange bar. (B, D, and G) Southern blots (from panels A, D, and F, respectively) of digested genomic DNA from the 3D7 parent parasite line, vector, and knockout clones, showing correct disruption of PfEH1 or PfEH2. Arrows indicate the expected size for correct integration, and asterisks indicate bands of unknown origin. (E and H) Growth of the relevant 3D7 parent and the single-knockout clones over 6 days. No differences were observed in growth between these knockout lines and 3D7 parents, with graphs representative of data from three independent experiments. The smooth curve is the fitted exponential growth equation. Panels F to H show data for a preliminary knockout of PfEH2 (at the stage of a nonclonal pool subjected once to positive and negative selection; provided by M. Klemba) which was previously published (89), generated using a vector containing the human dihydrofolate reductase cassette. This population was used to complete the generation of a second PfEH2 clonal knockout. To create the double-knockout line, the PfEH2 knockout (clone 11F) was transfected with the PfEH1 knockout vector containing the yDHODH selection cassette. Download Figure S3, JPG file, 1 MB

Table S1  InterPro domain information for the top five non-Apicomplexan hits from a PSI-BLAST pseudo-multiple-sequence alignment (comparing the P. falciparum [Pf] α/β-hydrolase sequence to a nonredundant protein data bank) generated using Phyre2 (30). Table S1, PDF file, 0.1 MB

Table S2  Putative interacting partners of PfEH1 and PfEH2 identified using mass spectrometry. GFP-tagged proteins were immunoprecipitated (using anti-GFP polyclonal antibody) from PfEH1-, PfEH2-, PfXL1-, PfXL2-GFP-tagged cultures or the 3D7 parent line, and the resulting proteins analyzed by mass spectrometry. Values are the numbers of unique peptide hits. The peptide counts for spectrin are shown in bold type. ND, no peptides detected. Table S2, PDF file, 0.1 MB

Table S3  Oligonucleotides used in this study. Restriction sites are underlined, InFusion overhangs are italicized, stop codons are shown in bold type, and mutations are shown in lowercase type. All oligonucleotides were purchased from Integrated DNA Technologies. Table S3, PDF file, 0.02 MB

Text S1  Supplemental Materials and Methods. Download Text S1, PDF file, 0.1 MB
