# Supplementary figures and images for: Exported Epoxide Hydrolases Modulate Erythrocyte Vasoactive Lipids during Plasmodium falciparum Infection
Source: mBio. 2016 Oct 18;7(5):e01538-16. doi: 10.1128/mBio.01538-16 (PMC5082902; doi:10.1128/mBio.01538-16)

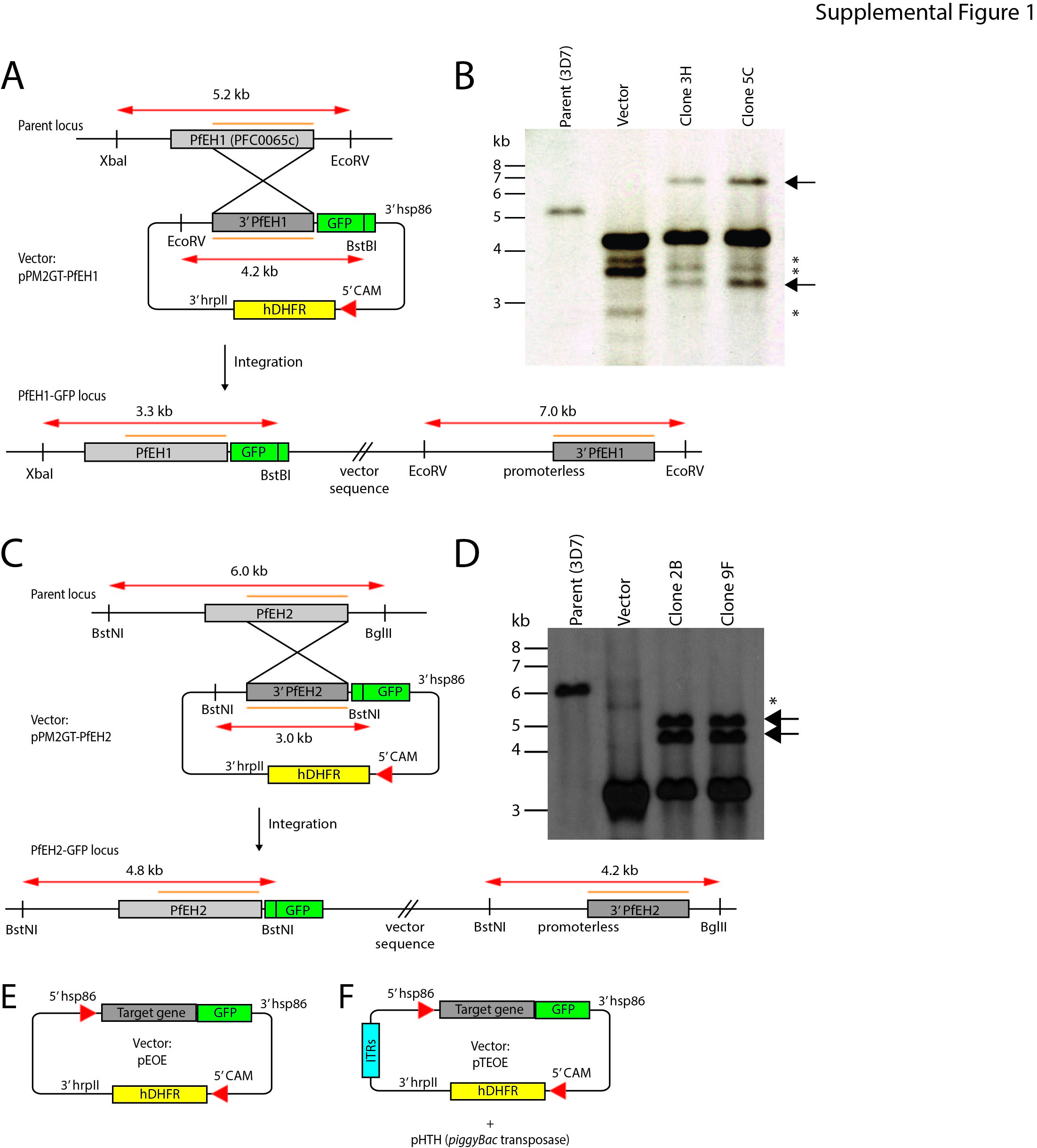

Supplement: Figure S1 — Generation of the GFP-tagged lines. (A) Schematic representation of the pEOE construct used to overexpress the protein of interest. The vector contains the human dihydrofolate reductase (hDHFR) positive-selection cassette, with selected parasites expressing the gene of interest under the strong, constitutive hsp86 promoter. (B) Schematic representation of the pTEOE construct used to overexpress the protein of interest via transposase-mediated integration. The vector contains the piggyBac element containing inverted terminal repeats (ITR) (90) and the hDHFR positive-selection cassette. The pHTH vector contains a cassette for transient expression of the piggyBac transposase (90), facilitating genomic integration of the pTEOE sequence, leading to stable expression of the gene of interest under the strong, constitutive hsp86 promoter. (C and E) Schema outlining the strategy for replacement of the 3′ region of the gene of interest by single-crossover homologous recombination. The pPM2GT vector (40) was used to append GFP to the 3′ end of the gene of interest, with positive selection mediated by the hDHFR selection cassette. The restriction digestion sites and the resulting expected sizes of DNA fragments that were utilized to characterize the locus by Southern blot analysis are labeled, with the probe represented by an orange bar. (D and F) Southern blots of digested genomic DNA from the 3D7 parent parasite line, vector, and tagged clones, showing correct integration of the GFP tag. Arrows indicate the expected size for correct integration, and asterisks indicate bands of unknown origin. In both PfEH1- and PfEH2-GFP lines, the plasmid band remains present, indicating integration of a concatemerized plasmid. Download [file mbo005163038sf1.jpg]

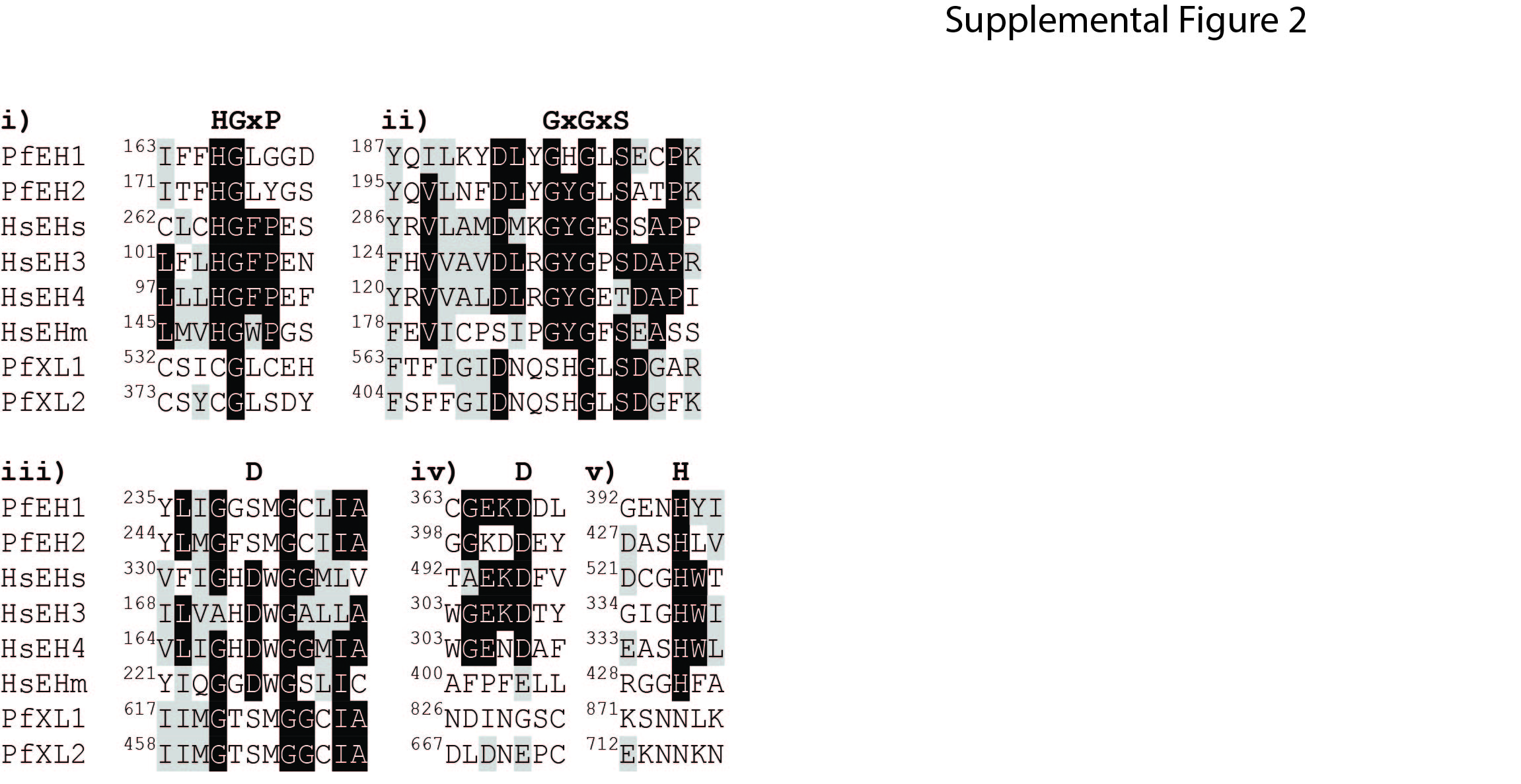

Supplement: Figure S2 — Amino acid alignment of PfEH1 and PfEH2 compared to known EH enzymes. Clustal Omega alignment of PfEH1, PfEH2, PfXL1, and PfXL2 with the equivalent regions in the four human EHs (Homo sapiens EHs [HsEHs]) (HsEH1, GenBank accession no. NP_001970.2; HsEH2, GenBank accession no. AAC41694.1; HsEH3,GenBank accession no. AAI15003.1; HsEH4, GenBank accession no.NP_775838.3). HsEH1 is the microsomal EH (HsEHm), and HsEH2 is the soluble EH (HsEhs). Identical residues are colored black, and functionally conserved residues are colored gray. The regions aligned represent the putative (i) oxyanion hole motif, (ii) GxGxS/T motif of unknown function, (iii) catalytic nucleophile, (iv) catalytic acid residue, and (v) catalytic base residue. Download [file mbo005163038sf2.jpg]

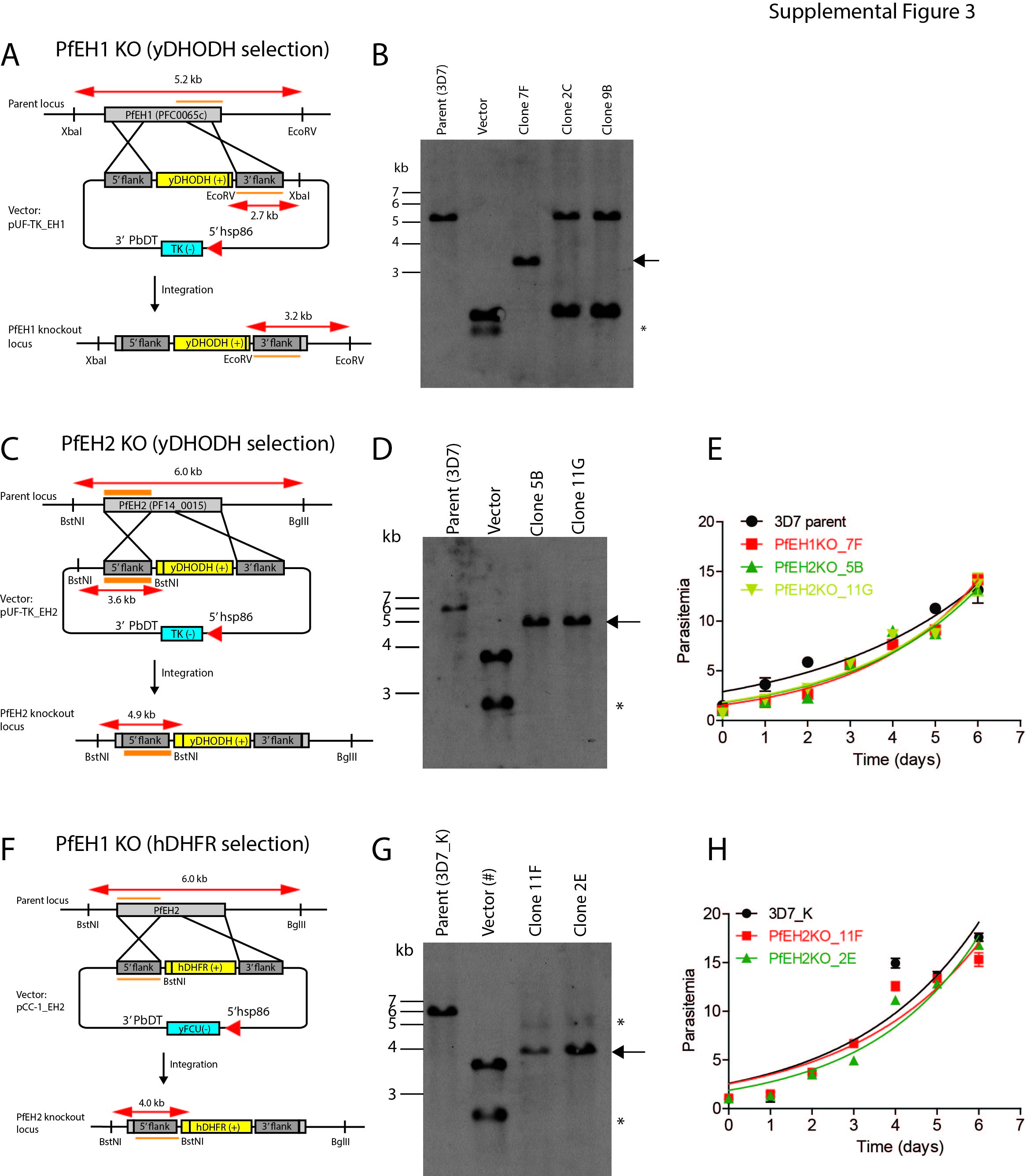

Supplement: Figure S3 — Generation of PfEH1 and PfEH2 single-knockout lines. (A, C, and F) Schema outlining the strategy for disruption of the gene of interest by double-crossover homologous recombination. The pUF-TK vector contains the yDHODH cassette for positive selection and the herpes simplex virus thymidine kinase (TK) cassette for negative selection. The pCC-1 vector contains the hDHFR cassette for positive selection and the yeast cytosine deaminase and uridyl phosphoribosyl transferase cassette for negative selection. The restriction digestion sites and the resulting expected sizes of DNA fragments that were utilized to characterize the locus by Southern blot analysis are labeled, with the probe represented by an orange bar. (B, D, and G) Southern blots (from panels A, D, and F, respectively) of digested genomic DNA from the 3D7 parent parasite line, vector, and knockout clones, showing correct disruption of PfEH1 or PfEH2. Arrows indicate the expected size for correct integration, and asterisks indicate bands of unknown origin. (E and H) Growth of the relevant 3D7 parent and the single-knockout clones over 6 days. No differences were observed in growth between these knockout lines and 3D7 parents, with graphs representative of data from three independent experiments. The smooth curve is the fitted exponential growth equation. Panels F to H show data for a preliminary knockout of PfEH2 (at the stage of a nonclonal pool subjected once to positive and negative selection; provided by M. Klemba) which was previously published (89), generated using a vector containing the human dihydrofolate reductase cassette. This population was used to complete the generation of a second PfEH2 clonal knockout. To create the double-knockout line, the PfEH2 knockout (clone 11F) was transfected with the PfEH1 knockout vector containing the yDHODH selection cassette. Download [file mbo005163038sf3.jpg]
