# Supplementary material for: Exported Epoxide Hydrolases Modulate Erythrocyte Vasoactive Lipids during Plasmodium falciparum Infection
Source: mBio. 2016 Oct 18;7(5):e01538-16. doi: 10.1128/mBio.01538-16 (PMC5082902; doi:10.1128/mBio.01538-16)
Supplement: Table S1 — InterPro domain information for the top five non-Apicomplexan hits from a PSI-BLAST pseudo-multiple-sequence alignment (comparing the P. falciparum [Pf] α/β-hydrolase sequence to a nonredundant protein data bank) generated using Phyre2 (30). [file mbo005163038st1.pdf]

Table 1: InterPro domain information for the top five non-Apicomplexan hits from a PSI-Blast pseudo-multiple sequence alignment (comparing the Pf α/β-hydrolase sequence to a non-redundant protein databank) generated using Phyre2 (7).

Input	E-value	Organism and gene ID (UniRef50 accession number)	IPRO29058 (α/β -hydrolase)	IPRO00639 (Epox_hydrolase_like)	IPRO22742 (Lysophopholipase_put/ serine aminopeptidase)
**PfEH1**	6.0E-62	*Desulfatibacillum alkenivorans* (Proteobacteria)α/β-hydrolase (B8FAJ5)	✓	✓	✗
	5.0E-60	*Nostoc punctiforme* (Cyanobacteria)α/β-hydrolase (B2IZH4)	✓	✓	✗
	4.0E-59	*Carnobacterium sp. AT7* (Firmicutes) Uncharacterized protein (A8UAB3)	✓	✓	✗
	7.0E-59	*Alpha proteobacterium* (Proteobacteria) putative 3-oxoadipate enol-lactonase (A8TT96)	✓	✗	✗
	8.0E-59	*Pseudoalteromonas atlantica* (strain T6c) (Proteobacteria) α/β-hydrolase (Q15ZT2)	✓	✓	✗
**PfEH2**	1.0E-70	*Homo sapiens* (Human) Soluble bifunctional epoxide hydrolase EPHX2 (P34913)	✓	✓	✗
	1.0E-68	*Sus scrofa* (Pig) Soluble bifunctional epoxide hydrolase EPHX2 (Q6Q2C2)	✓	✓	✗
	9.0E-65	*Parvibaculum lavamentivorans* (Proteobacterium) α/β-hydrolase (A7HW23)	✓	✓	✗
	3.0E-63	*Salmo salar* (Atlantic salmon) Epoxide hydrolase 2 (P34914)	✓	✓	✗
	5.0E-62	*Nostoc punctiforme* (Cyanobacteria)α/β-hydrolase (B2IV02)	✓	✓	✗
**PfXL1**	1.0E-57	*Salinibacter ruber* (Bacteroidetes) Lysophospholipase (Q2S1I8)	✓	✗	✓
	2.0E-57	*Herpetosiphon aurantiacus* (Chloroflexi) α/β-hydrolase (A9B3M2)	✓	✗	✓
	2.0E-57	*Chlamydomonas reinhardtii* (green algae) Predicted protein (A8IJR0)	✓	✗	✗
	1.0E-55	*Oryza sativa subsp. japonica* (rice) (Q0J1Y4)	✓	✗	✓
	6.0E-55	*Desulfococcus oleovorans* (Proteobacteria)α/β-hydrolase (A8ZXX5)	✓	✗	✓
**PfXL2**	3.0E-55	*Salinibacter ruber* (Bacteroidetes) Lysophospholipase (Q2S1I8)	✓	✗	✓
	8.0E-54	*Geodermatophilus obscurus* (Actinobacteria) α/β-hydrolase (D2S453)	✓	✗	✗
	1.0E-53	*Oryza sativa subsp. japonica* (rice) Lysophospholipase (Q8H4S9)	✓	✗	✗
	4.0E-53	*Gordonia bronchialis* (Actinobacteria) Acylglycerol lipase (D0L785)	✓	✗	✗
	6.0E-52	*Herpetosiphon aurantiacus* (Chloroflexi)α/β-hydrolase (A9B3M2)	✓	✗	✓
