# Supplementary material for: Exported Epoxide Hydrolases Modulate Erythrocyte Vasoactive Lipids during Plasmodium falciparum Infection
Source: mBio. 2016 Oct 18;7(5):e01538-16. doi: 10.1128/mBio.01538-16 (PMC5082902; doi:10.1128/mBio.01538-16)
Supplement: Table S2 — Putative interacting partners of PfEH1 and PfEH2 identified using mass spectrometry. GFP-tagged proteins were immunoprecipitated (using anti-GFP polyclonal antibody) from PfEH1-, PfEH2-, PfXL1-, PfXL2-GFP-tagged cultures or the 3D7 parent line, and the resulting proteins analyzed by mass spectrometry. Values are the numbers of unique peptide hits. The peptide counts for spectrin are shown in bold type. ND, no peptides detected. [file mbo005163038st2.pdf]

Supplemental Table 2: Putative interacting partners of PfEH1 and PfEH2 identified using mass-spectrometry. GFP-tagged proteins were immunoprecipitated (using anti-GFP polyclonal antibody) from PfEH1-, PfEH2-, PfXL1-, PfXL2- GFP tagged cultures, or the 3D7 parent line and the resulting proteins analysed by mass spectrometry. Values are the number of unique peptide hits. The peptide counts for spectrin are bolded. ND = no peptides detected.

	**3D7 parent**	**PfEH1-GFP (clone 3H)**	**PfEH2-GFP (clone 2B)**	**PfXL1-GFP (TEOE pool)**	**PfXL2-GFP (TEOE pool)**
Bait	ND	14	21	21	53
Human proteins					
α-spectrin	**5**	**75**	**97**	**7**	**22**
β-spectrin	**5**	**65**	**83**	**2**	**11**
ankyrin	7	38	46	8	21
Solute carrier family 4 AE1	8	16	17	15	17
Carbonic anhydrase 1	ND	9	6	11	14
Protein 4.1	2	5	7	1	1
Parasite proteins					
HSP70 (PF3D7_0818900)	34	38	34	35	34
PV1	23	23	24	27	23
HSP101	ND	10	16	22	32
PTEX150	ND	2	9	7	4
RESA	2	15	15	8	14
MESA	2	17	41	11	24
