# Supplementary material for: Exported Epoxide Hydrolases Modulate Erythrocyte Vasoactive Lipids during Plasmodium falciparum Infection
Source: mBio. 2016 Oct 18;7(5):e01538-16. doi: 10.1128/mBio.01538-16 (PMC5082902; doi:10.1128/mBio.01538-16)
Supplement: Table S3 — Oligonucleotides used in this study. Restriction sites are underlined, InFusion overhangs are italicized, stop codons are shown in bold type, and mutations are shown in lowercase type. All oligonucleotides were purchased from Integrated DNA Technologies. [file mbo005163038st3.pdf]

Supplemental Table 3: Oligonucleotides used in this study. Restriction sites are underlined, InFusion overhangs are italicized, stop codons are bolded and mutations are in lower case. All oligonucleotides were purchased from Integrated DNA Technologies.

Oligonucleotide ID	Sequence
**PfEH1/2 into pPM2GT:**	
NJS1	*CACTATAGAACTCGAG*GATGGTGCTGAAAAGATATG
NJS2	*TGTGCTGCACCTGGCCTAGG*TGTGGTACATCCATTTTTATC
NJS4	*CACTATAGAACTCGAG*GAATGCCCTTATGAATACGG
NJS5	*TGTGCTGCACCTGGCCTAGG*TGTTTTATATTGACCATTTTTATC
**PfEH1/2 into pEOE and pTEOE:**	
NJS160	*CGATTTTTTCTCGAG*ATGAAAAAAGGAATGAAGAAATC
NJS161	*ATGTGCTGCACCTGGCCTAGG*TGTGGTACATCCATTTTTATC
NJS162	*CGATTTTTTCTCGAG*ATGAGAAATATTAATGGGTTC
NJS163	*ATGTGCTGCACCTGGCCTAGG*TGTTTTATATTGACCATTTTTATC
NJS299	*CGATTTTTTCTCGAG*ATGTCAGTTCACGAAAGGATACG
NJS300	*ATGTGCTGCACCTGGCCTAGG*TACAAATATATTATTTATCCAGTCAG
NJS301	*CGATTTTTTCTCGAG*ATGCTCAATTTTAGGAGGTGGAG
NJS302	*ATGTGCTGCACCTGGCCTAGG*TGGATATATATTATTAAGCC
**Mutagenesis of pTEOE_EH1:**	
NJS524	GAGGAAATTAAATTAAGATATTTAAGTGAAATTGAAAAAAATGATATATGTG
NJS525	GAGGAAATTAAATTAAGATATTTAAGTGAAGATGCATATGATGGTGCTG
NJS526	GAGGAAATTAAATTAAGATATTTAAGTGAAACACCAAAATTTTTACTGGATAG
NJS527	GAGGAAATTAAATTAAGATATTTAAGTGAACCTAGGCCAGGTGCAGCACAT
NJS528	GAATATAAGGAAAAATGTTACATTAAGTATGAACCTAGGCCAGGTGCAGCACAT
**PfEH1/2 into pET28a:**	
NJS49	*GTGCCGCGCGGCAGCCATATG*ACGGAATTAAATAAGAGGTC
NJS50	*GTGGTGGTGGTGCTCGAG***CTA**TGTGGTACATCCATTTTTATC
NJS55	*GTGCCGCGCGGCAGCCATATG*GTAATTAAAGATGGTATAGG
NJS56	*GTGGTGGTGGTGCTCGAG***CTA**TGTTTTATATTGACCATTTTTATC
NJS219	*GAAGGAGATATACCATGG*GAAGTGAAACGGAATTAAATAAGAGGTC
NJS220	*GTGGTGGTGGTGCTCGAG*TGTGGTACATCCATTTTTATC
NJS221	*GAAGGAGATATACCATGG*GATGTGATGTAATTAAAGATGGTATAGG
NJS222	*GTGGTGGTGGTGCTCGAG*TGTTTTATATTGACCATTTTTATC
**Mutagenesis of pET28a_EH1/2:**	
NJS124	GGAATTTTATTTGATTGGTGGTgCAATGGGTTGTTTAATAGCTGC
NJS 125	GCAGCTATTAAACAACCCATTGcACCACCAATCAAATAAAATTCC
NJS 128	CCTACTTTAATATTTTGTGGAGAAAAAGcTGATCTTTATGATGACGATG
NJS 129	CATCGTCATCATAAAGATCAgCTTTTTCTCCACAAAATATTAAAGTAGG
NJS 130	CATATTATTGTTTTCAAAGGAGAAAATgcTTATATAATACCTTCAAGAAC
NJS 131	GTTCTTGAAGGTATTATATAAgcATTTTCTCCTTTGAAAACAATAATATG
NJS134	GGAACATATGGAATAACAAATGCTGAACTTATTGGAAATAAAGACAAC
NJS 135	GTTGTCTTTATTTCCAATAAGTTCAGCATTTGTTATTCCATATGTTCC
NJS170	GGAATTTTATTTGATTGGTGGTgatATGGGTTGTTTAATAGCTGC

NJS171	GCAGCTATTAAACAACCCATatcACCACCAATCAAATAAAATTCC
**PfEH1/2 into pUF-TK:**	
NJS245	*CAGGCGCCAGCCTAGG*CATATGTTTTGGTTTGTATTG
NJS246	*ATCGATAACTCCATGG*CTTTATCTCCACCAAGCCCATG
NJS247	*AGATCTTCGGACTAGT*GATTGGTGGTTCAATGGGTTG
NJS248	*CAATGGCCCCTTTCCGCGG*GTACATCCATTTTTATCAACAG
NJS249	*CAGGCGCCAGCCTAGG*CTTAAGTACATATGATAAAC
NJS250	*ATCGATAACTCCATGG*CTCAGATTCCGGATTCCCCTTC
NJS251	*AGATCTTCGGACTAGT*CATTTGAACCTAGAAAATAAGG
NJS252	*CAATGGCCCCTTTCCGCGG*GTAAACATACATCATTGGGCG
**qRT-PCR:**	
EH1_primer1	TGGTGGTTCAATGGGTTGT
EH1_primer2	CCTAACATACCCACAGGTGATAAA
EH1_probe	5’ 6-FAM/AGCTGCTGC/ZEN/ATTTGCACAGAAAT/3’ IABkFQ
EH2_primer1	CACAATCCATACTTGGTTGCATAA
EH2_primer2	CCTCTGAACAATATTCATCATCCTTTC
EH2_probe	5’ 6-FAM/TTTGCATAT/ZEN/GTGGAGTGCTCATCA/3’ IABkFQ
Actin_primer1	TGTTGACAACGGATCAGGTAAT
Actin_primer2	CCTGGATTCTTTGGTCTTCCTAC
Actin_probe	5’ 6-FAM/AGGAGTTGC/ZEN/AGGAGATGATGCACC/3’ IABkFQ

\
