# Supplementary material for: Exported Epoxide Hydrolases Modulate Erythrocyte Vasoactive Lipids during Plasmodium falciparum Infection
Source: mBio. 2016 Oct 18;7(5):e01538-16. doi: 10.1128/mBio.01538-16 (PMC5082902; doi:10.1128/mBio.01538-16)
Supplement: Text S1 — Supplemental Materials and Methods. Download [file mbo005163038s1.pdf]

SUPPLEMENTAL INFORMATION for

Exported epoxide hydrolases modulate erythrocyte vasoactive lipids during *Plasmodium falciparum* infection

Natalie J. Spillman [a,b], Varun K. Dalmia [a], Daniel E. Goldberg [a]

Departments of Medicine and Molecular Microbiology, Washington University School of Medicine, St. Louis, Missouri, USA [a]; Research School of Biology, The Australian National University, Canberra, Australian Capital Territory, Australia[b]

Supplemental Contains:

Supplemental Materials and Methods

Supplemental Figure Legends

Supplemental References

Supplemental Table 1

Supplemental Table 2

Supplemental Table 3

Supplemental Figure 1

Supplemental Figure 2

Supplemental Figure 3

Supplemental Materials and Methods

Growth assays and transfection

To compare the growth rates of several cultures over multiples days, asynchronous cultures were adjusted to 0.5 % parasitemia in 4 mL culture volume. Parasitemia was measured daily by flow cytometry (BD FACSCanto flow cytometer; BD Biosystems) using 10 µL aliquots of culture stained with 1.5 µg/mL acridine orange in PBS (Molecular Probes, Invitrogen). Growth rates were calculated by fitting a non-linear curve using the expression Parasitemia = y_o^{k*Time} where y_0 is the initial parasitemia (time = 0) and k is the rate constant (units of times; day^{-1}).

Parasites were transfected with 100 µg of supercoiled plasmid as described elsewhere (1). Positive selection was applied 24-48 hours post transfection with the appropriate pressure- either 10 nM WR99210 (human DHFR selection cassette) or 2 µM DSM-1 (yeast DHODH selection cassette). Cultures were enriched for integration events (loss of the episomal plasmids) by two rounds of off-on drug cycling. Ganciclovir (10 µM; Herpes simplex virus thymidine kinase selection cassette) was used for three weeks of negative selection during creation of the knockout lines. Clones were attained by limiting dilution and genotypes were confirmed by Southern blot using the Amersham AlkPhos Direct Labeling (GE Healthcare) as described previously (2). Genomic DNA was isolated using the Qiagen QIAamp Blood Mini Kit.

Plasmid Construction

Restriction enzymes were purchased from New England Biolabs and cloning was performed using InFusion (Clontech). All plasmids were verified by analytical digest and Sanger sequencing. All oligonucleotides used in this study are listed in Supplemental Table 3.

To overexpress a C-terminal GFP-tagged protein, where expression was under control of the HSP86 promoter, full length sequences (amplified from 3D7 genomic DNA) were inserted into the XhoI/AvrII sites of the pEOE (3) vector ('Episomal OverExpression') using primers NJS160&161 (PfEH1), NJS162&163 (PfEH2). The resultant parasites (uncloned pool) were used to generate data presented in Figures 1(A,B), 2(C-F) and 6.

To overexpress PfXL1 and PfXL2 (C-terminal GFP-tag, under expression of the HSP86 promoter), full length sequences were inserted into the XhoI/AvrII sites of the pTEOE vector (4) using primers NJS299&300 (PfXL1) and NJS301&302 (PfXL2). pTEOE constructs were cotransfected with 50 µg pHTH (MRA-912; deposited at MR4 by Prof. John Adams, as part of the BEI Resources Repository, NIAID, NIH). The resultant parasites (uncloned pool) were used to generate data presented in Figures 1(C,D) and Supplemental Table 2.

To create truncation mutations of PfEH1, the full length sequence (amplified from 3D7 genomic DNA) were inserted into the XhoI/AvrII sites of the pTEOE vector using primers NJS160&161. Site-directed mutagenesis (QuikChange Lightning; Agilent Technologies) was used to generate truncation mutants (CR1 with primer NJS524, CR2 with NJS525, CR3 with NJS526, EH1Trunc with NJS527 and ABHTrunc with NJS528). The resultant parasites (uncloned pool) were used to generate data presented in Figure 3.

To create a C-terminal GFP-tagged PfEH1 or PfEH2, where expression was under the control of the endogenous promoter, the pPM2GT vector (5) was used. Primers NJS1&2 were used to amplify a ~1000 bp flank of PfEH1 from 3D7 genomic DNA, and the resulting amplicon was inserted into the XhoI/AvrII sites of pPM2GT to create pPM2GT_PfEH1. Primers NJS4&5 were used for the construction of pPM2GT_PfEH2. The resultant parasites were cloned by limiting dilution. Clones 3H and 5C (PfEH1) and clones 2B and 9F (PfEH2) were used to generate data presented in Figures 2(A-D), Supplemental Table 2 and Supplemental Figure 1.

To knock out PfEH1 or PfEH2, two flanks were amplified from 3D7 genomic DNA and inserted into the pUF-TK vector using the AvrII/NcoI sites for the 5' flank and the SpeI/SacII sites for the 3' flank. The pUF-TK vector is the pUF vector (6), containing the yDHODH positive selection cassette instead of the hDHFR selection cassette. Primers NJS245&246 and NJS247&248 were used for construction of pUF-TK_PfEH1 and NJS249&250 and NJS251&252 used for construction of pUF-TK_PfEH2. The resultant parasites were cloned by limiting dilution. Clones 7F (PfEH1 individual knockout), clones 5B and 11G (PfEH2 individual knockout) and clones 11F and 2E (PfEH1+2 double knockout) were used to generate data presented in Figures 5, 6 and Supplemental Figure 3.

For recombinant protein expression, primers NJS49&50 were used to amplify PfEH1 (to express the protein beginning residue 58 (after PEXEL motif)) from 3D7 genomic DNA. Primers NJS55&56 were used to amplify PfEH2 (beginning residue 93 (after PEXEL motif)). To introduce an N-terminal 6xHis tag the amplicons were inserted into the NdeI/XhoI sites of the pET28a vector. To introduce the C-terminal 6xHis tag amplicons were inserted into the NcoI/XhoI sites of the pET28a vector using primers NJS219&220 to amplify the PfEH1 flank and primers NJS221&222 to amplify the PfEH2 flank. Site-directed mutagenesis (QuikChange II; Agilent Technologies) was used to generate mutants of PfEH1 (S240A with primers NJS124&125; D367A, NJS128&129; H395A, NJS130&131; S207A, NJS134&135; S240D, NJS170&171).

Bioinformatic analyses

A PSI-Blast pseudo-multiple sequence alignment (comparing the protein sequence of interest to a non-redundant protein databank) was generated using the online Phyre2 server (7). InterPro domain information was extracted from the UniProtKB database (8). Local pairwise alignments,

identity/similarity calculations and Dayhoff Statistics (PEPSTAT) were performed/calculated using the EMBOSS Matcher online server (9). Multiple sequence alignment was performed using the Clustal Omega (9) online server and alignment appearance was adjusted using Boxshade on the Mobyle@Pasteur website (http://mobyle.pasteur.fr/cgi-bin/portal.py?#forms::boxshade). A homology model of PfEH1was generated using the Robetta server (10) and visualized using the PyMOL Molecular Graphics System, Version 1.7.4 (Schrödinger, LLC). The top template structures used for modelling were the *Pseudomonas putida* α/β-hydrolase (PDB:1ZOI) and human soluble epoxide hydrolase (HsEH2; PDB:1S8O).

Indirect Immunofluorescence assay (IFA) and Microscopy

Indirect IFA was performed as previously described (11, 12). Briefly, cells were settled onto coverslips coated with 0.5 mg/mL concanavalin A and fixed in 4 % (v/v) paraformaldehyde/ 0.0075 % (v/v) glutaraldehyde in PBS for 30 min. Cells were permeabilized with 0.1 % Triton (10 min), treated with 0.1 mg/mL $NaBH_4$ (10 min) before blocking in 3 % (w/v) bovine serum albumin (1-16 hr). Concentrations of the primary antibodies used were: rabbit polyclonal anti-GFP (ab6556 Abcam) at 1:200, mouse anti-RESA mAb clone 28/2 ((13) kindly provided by Robin Anders, La Trobe University) at 1:600. Secondary antibodies (Alexa Fluor 488-conjugated goat anti-rabbit or Alexa Fluor 594 conjugated goat anti-mouse) were used at 1:2000. Coverslips were mounted in Prolong Gold Antifade Mountant with DAPI (Molecular Probes). Fixed cells were visualized using a AxioImager.M1 epifluorescence microscope (Zeiss) equipped with Hamamatsu ORCA-ER digital CCD camera. Acquisition of image stacks and deconvolution was performed as previously described (11). Images were processed using AxioVision software Release 4.8.1.

Immunoprecipitation

For immunoprecipitation, enriched, infected erythrocytes were lysed in 0.1% Triton X-100 in PBS with Protease Inhibitor Cocktail (Roche). After nutating for 1 hr at 4 °C the lysates were clarified by centrifugation at 16,000 g for 5 minutes. The supernatant was pre-cleared by nutating with Protein A Dynabeads (Molecular Probes) for 3 hrs (4 °C). The pre-clear beads were discarded and the lysate nutated with rabbit polyclonal anti-GFP (ab6556 Abcam; 2-5 µg) for 16 hrs (4 °C). The antibody/lysate mix was added to the Dynabeads (3 hrs; 4 °C) and purified protein complexes were isolated after five washes in 0.1% Triton X-100 in PBS.

Western blotting

Western blotting was performed using rabbit polyclonal anti-GFP (ab6556 Abcam; 1:10,000), rabbit polyclonal anti-PfHAD1 (14) (1:20,000; provided by A. Odom), rabbit polyclonal anti-spectrin (S1515 Sigma-Aldrich; 1:400), rabbit polyclonal anti-PfSERP(15) (1:1000; provided by J.

Przyborski) and mouse monoclonal anti-PfHRPII (16) (1:1000; provided by D. Taylor). IR680- or 800-conjugated secondary antibodies were used at 1:15,000 with the LI-COR Odyssey Imaging System (11). Quantification was performed using the LI-COR Odyssey Image Studio software v4.

Mass Spectrometry and peptide identification

Immunoprecipitation samples were boiled in SDS sample buffer (5 min; 95 °C) and briefly (10 min) run into a 12.5% acrylamide gel. Samples were excised and shipped on dry ice to the 'FingerPrints' Proteomics and Mass Spectrometry Facility at the University of Dundee. Samples were trypsin digested in-gel and peptides were analysed by nano-LC ESI/MS/MS ('Standard' service on LTQ Orbitrap XL). Peptides were queried against human (International Protein Index) and *Plasmodium* (PlasmoDB) protein databases.

Recombinant expression of PfEH1 and 2

PfEH1 was expressed in BL21(DE3) *E. coli* and PfEH2 expressed in BL21-CodonPlus(DE3)-RIPL *E. coli* as previously described (17), with several modifications. For PfEH1 expression, after an OD600 of 0.6 was reached the culture was cooled to 16 °C, induced with 1 mM IPTG, and was grown for 20 hr at 16 °C. For PfEH2 expression, after an OD600 of 0.6 was reached the culture remained at 37 °C, was induced with 1 mM IPTG, and was grown for 2.5 hr at 37 °C. The bacterial pellets were harvested, clarified, and purified using a 1 mL HisTrap HP affinity column (GE Healthcare) followed by size exclusion chromatography (24 mL Superdex 200 gel filtration column) as described previously (17). Protein concentration was determined using the BCA Protein Assay kit (Pierce) using the microplate procedure.

Supplemental Figure Legends:

Supplemental Figure 1: Generation of the GFP-tagged lines. (A) Schematic representation of the pEOE(4) construct used to overexpress the protein of interest. The vector contains the hDHFR positive selection cassette, with selected parasites expressing the gene of interest under the strong, constitutive hsp86 promoter. (B) Schematic representation of the pTEOE construct used to overexpress the protein of interest *via* transposase-mediated integration. The vector contains the *piggyBac* element containing inverted terminal repeats (ITR)(18) and the hDHFR positive selection cassette. The pHTH vector contains a cassette for transient expression of the *piggyBac* transposase (18), facilitating genomic integration of the pTEOE sequence, leading to stable expression of the gene of interest under the strong, constitutive hsp86 promoter. (C) and (E) are schema outlining the strategy for replacement of the 3' region of the gene of interest by single crossover homologous

recombination. The pPM2GT vector (5) was used to append GFP to the 3' end of the gene of interest, with positive selection mediated by the hDHFR selection cassette. The restriction digest sites, and resulting expected sizes that were utilized to characterize the locus by Southern blot analysis are labelled, with the probe represented by an orange bar. (D) and (F) are Southern blots of digested genomic DNA from the 3D7 parent parasite line, vector and tagged clones, showing correct integration of the GFP tag. Arrows indicate the expected size for correct integration and asterisks indicate bands of unknown origin. In both PfEH1- and PfEH2-GFP lines the plasmid band remains present, indicating integration of a concatemerized plasmid.

Supplemental Figure 2: Amino acid alignment of PfEH1 and PfEH2 compared to known EH enzymes. Clustal Omega alignment of PfEH1, PfEH2, PfXL1, PfXL2 with the equivalent regions in the four human EHs (Accession codes: HsEH1, NP_001970.2; HsEH2, AAC41694.1; HsEH3, AAI15003.1; HsEH4, NP_775838.3). HsEH1 is the microsomal (m) EH and HsEH2 is the soluble (s) EH. Identical residues are colored black and functionally conserved residues are colored gray. The regions aligned represent the putative i) oxyanion hole motif, ii) GxGxS/T motif of unknown function, iii) catalytic nucleophile, iv) catalytic acid residue and v) catalytic base residue.

Supplemental Figure 3: Generation of PfEH1 and PfEH2 single knockout lines. (A), (C) and (F) are schema outlining the strategy for disruption of the gene of interest by double crossover homologous recombination. The pUF-TK vector contains the yDHODH cassette for positive selection and the *Herpes simplex* virus thymidine kinase cassette for negative selection. The pCC-1 vector contains the hDHFR cassette for positive selection and the yeast cytosine deaminase and uridyl phosphoribosyl transferase cassette for negative selection. The restriction digest sites, and resulting expected sizes that were utilized to characterize the locus by Southern blot analysis are labelled, with the probe represented by an orange bar. (B), (D) and (G) are Southern blots (from A) D) and F) respectively) of digested genomic DNA from the 3D7 parent parasite line, vector and knock out clones, showing correct disruption of PfEH1 or 2. Arrows indicate the expected size for correct integration and asterisks indicate bands of unknown origin. (E) and (H) Growth of the relevant 3D7 parent, and the single knock out clones, over six days. No differences were observed in growth between these knockout lines and 3D7 parents, with graphs representative of data from n =3 independent experiments. The smooth curve is the fitted exponential growth equation. (F)-(H) are based on a preliminary knockout of PfEH2 (at the stage of an unclonal pool cycled once on positive and negative selection; provided by M. Klemba) which was previously published (19), generated using a vector containing the human dihydrofolate reductase cassette. This population was used to complete the generation of a second PfEH2 clonal knockout. To create the double knockout line, the PfEH2 knockout (clone 11F) was transfected with the PfEH1 knockout vector containing the yDHODH selection cassette.

Supplemental References:

1. **Rug M, Maier AG.** 2013. Transfection of *Plasmodium falciparum*. Methods Mol Biol **923:**75-98.
2. **Ponpuak M, Klemba M, Park M, Gluzman IY, Lamppa GK, Goldberg DE.** 2007. A role for falcilysin in transit peptide degradation in the *Plasmodium falciparum* apicoplast. Mol Microbiol **63:**314-334.
3. **Russo I, Oksman A, Goldberg DE.** 2009. Fatty acid acylation regulates trafficking of the unusual *Plasmodium falciparum* calpain to the nucleolus. Mol Microbiol **72:**229-245.
4. **Pal P, Daniels BP, Oskman A, Diamond MS, Klein RS, Goldberg DE.** 2016. Plasmodium falciparum Histidine-Rich Protein II Compromises Brain Endothelial Barriers and May Promote Cerebral Malaria Pathogenesis. MBio **7**.
5. **Klemba M, Beatty W, Gluzman I, Goldberg DE.** 2004. Trafficking of plasmepsin II to the food vacuole of the malaria parasite *Plasmodium falciparum*. J Cell Biol **164:**47-56.
6. **Ganesan SM, Morrisey JM, Ke H, Painter HJ, Laroiya K, Phillips MA, Rathod PK, Mather MW, Vaidya AB.** 2011. Yeast dihydroorotate dehydrogenase as a new selectable marker for Plasmodium falciparum transfection. Mol Biochem Parasitol **177:**29-34.
7. **Kelley LA, Mezulis S, Yates CM, Wass MN, Sternberg MJ.** 2015. The Phyre2 web portal for protein modeling, prediction and analysis. Nat Protoc **10:**845-858.
8. **UniProt C.** 2015. UniProt: a hub for protein information. Nucleic Acids Res **43:**D204-212.
9. **Li W, Cowley A, Uludag M, Gur T, McWilliam H, Squizzato S, Park YM, Buso N, Lopez R.** 2015. The EMBL-EBI bioinformatics web and programmatic tools framework. Nucleic Acids Res **43:**W580-584.
10. **Kim DE, Chivian D, Baker D.** 2004. Protein structure prediction and analysis using the Robetta server. Nucleic Acids Res **32:**W526-531.
11. **Beck JR, Muralidharan V, Oksman A, Goldberg DE.** 2014. PTEX component HSP101 mediates export of diverse malaria effectors into host erythrocytes. Nature **511:**592-595.
12. **Tonkin CJ, van Dooren GG, Spurck TP, Struck NS, Good RT, Handman E, Cowman AF, McFadden GI.** 2004. Localization of organellar proteins in *Plasmodium falciparum* using a novel set of transfection vectors and a new immunofluorescence fixation method. Mol Biochem Parasitol **137:**13-21.
13. **Anders RF, Barzaga N, Shi PT, Scalon DB, Brown LE, Thomas LM, Brown GV, Stahl HD, Coppel RL, Kemp DJ.** 1986. Molecular strategies of parasitic invasion. In: Molecular Strategies of Parasitic Invasion. UCLA Symposium of Molecular and Cellular Biology. . Journal of Cellular Biochemistry **32:**111-174.
14. **Guggisberg AM, Park J, Edwards RL, Kelly ML, Hodge DM, Tolia NH, Odom AR.** 2014. A sugar phosphatase regulates the methylerythritol phosphate (MEP) pathway in malaria parasites. Nat Commun **5:**4467.
15. **Ragge K, Arnold HH, Tummler M, Knapp B, Hundt E, Lingelbach K.** 1990. In vitro biosynthesis and membrane translocation of the serine rich protein of *Plasmodium falciparum*. Mol Biochem Parasitol **42:**93-100.
16. **Parra ME, Evans CB, Taylor DW.** 1991. Identification of *Plasmodium falciparum* histidine-rich protein 2 in the plasma of humans with malaria. J Clin Microbiol **29:**1629-1634.
17. **Sigala PA, Crowley JR, Hsieh S, Henderson JP, Goldberg DE.** 2012. Direct tests of enzymatic heme degradation by the malaria parasite *Plasmodium falciparum*. J Biol Chem **287:**37793-37807.
18. **Balu B, Shoue DA, Fraser MJ, Jr., Adams JH.** 2005. High-efficiency transformation of *Plasmodium falciparum* by the lepidopteran transposable element piggyBac. Proc Natl Acad Sci U S A **102:**16391-16396.
19. **Denloye T, Dalal S, Klemba M.** 2012. Characterization of a glycerophosphodiesterase with an unusual tripartite distribution and an important role in the asexual blood stages of *Plasmodium falciparum*. Mol Biochem Parasitol **186:**29-37.

Table 1: InterPro domain information for the top five non-Apicomplexan hits from a PSI-Blast pseudo-multiple sequence alignment (comparing the Pf α/β-hydrolase sequence to a non-redundant protein databank) generated using Phyre2 (7).

Input	E-value	Organism and gene ID (UniRef50 accession number)	IPRO29058 (α/β -hydrolase)	IPRO00639 (Epox_hydrolase_like)	IPRO22742 (Lysophopholipase_put/ serine aminopeptidase)
**PfEH1**	6.0E-62	*Desulfatibacillum alkenivorans* (Proteobacteria)α/β-hydrolase (B8FAJ5)	✓	✓	✗
	5.0E-60	*Nostoc punctiforme* (Cyanobacteria)α/β-hydrolase (B2IZH4)	✓	✓	✗
	4.0E-59	*Carnobacterium sp. AT7* (Firmicutes) Uncharacterized protein (A8UAB3)	✓	✓	✗
	7.0E-59	*Alpha proteobacterium* (Proteobacteria) putative 3-oxoadipate enol-lactonase (A8TT96)	✓	✗	✗
	8.0E-59	*Pseudoalteromonas atlantica* (strain T6c) (Proteobacteria) α/β-hydrolase (Q15ZT2)	✓	✓	✗
**PfEH2**	1.0E-70	*Homo sapiens* (Human) Soluble bifunctional epoxide hydrolase EPHX2 (P34913)	✓	✓	✗
	1.0E-68	*Sus scrofa* (Pig) Soluble bifunctional epoxide hydrolase EPHX2 (Q6Q2C2)	✓	✓	✗
	9.0E-65	*Parvibaculum lavamentivorans* (Proteobacterium) α/β-hydrolase (A7HW23)	✓	✓	✗
	3.0E-63	*Salmo salar* (Atlantic salmon) Epoxide hydrolase 2 (P34914)	✓	✓	✗
	5.0E-62	*Nostoc punctiforme* (Cyanobacteria)α/β-hydrolase (B2IV02)	✓	✓	✗
**PfXL1**	1.0E-57	*Salinibacter ruber* (Bacteroidetes) Lysophospholipase (Q2S1I8)	✓	✗	✓
	2.0E-57	*Herpetosiphon aurantiacus* (Chloroflexi) α/β-hydrolase (A9B3M2)	✓	✗	✓
	2.0E-57	*Chlamydomonas reinhardtii* (green algae) Predicted protein (A8IJR0)	✓	✗	✗
	1.0E-55	*Oryza sativa subsp. japonica* (rice) (Q0J1Y4)	✓	✗	✓
	6.0E-55	*Desulfococcus oleovorans* (Proteobacteria)α/β-hydrolase (A8ZXX5)	✓	✗	✓
**PfXL2**	3.0E-55	*Salinibacter ruber* (Bacteroidetes) Lysophospholipase (Q2S1I8)	✓	✗	✓
	8.0E-54	*Geodermatophilus obscurus* (Actinobacteria) α/β-hydrolase (D2S453)	✓	✗	✗
	1.0E-53	*Oryza sativa subsp. japonica* (rice) Lysophospholipase (Q8H4S9)	✓	✗	✗
	4.0E-53	*Gordonia bronchialis* (Actinobacteria) Acylglycerol lipase (D0L785)	✓	✗	✗
	6.0E-52	*Herpetosiphon aurantiacus* (Chloroflexi)α/β-hydrolase (A9B3M2)	✓	✗	✓

Supplemental Table 2: Putative interacting partners of PfEH1 and PfEH2 identified using mass-spectrometry. GFP-tagged proteins were immunoprecipitated (using anti-GFP polyclonal antibody) from PfEH1-, PfEH2-, PfXL1-, PfXL2- GFP tagged cultures, or the 3D7 parent line and the resulting proteins analysed by mass spectrometry. Values are the number of unique peptide hits. The peptide counts for spectrin are bolded. ND = no peptides detected.

	**3D7 parent**	**PfEH1-GFP (clone 3H)**	**PfEH2-GFP (clone 2B)**	**PfXL1-GFP (TEOE pool)**	**PfXL2-GFP (TEOE pool)**
Bait	ND	14	21	21	53
Human proteins					
α-spectrin	**5**	**75**	**97**	**7**	**22**
β-spectrin	**5**	**65**	**83**	**2**	**11**
ankyrin	7	38	46	8	21
Solute carrier family 4 AE1	8	16	17	15	17
Carbonic anhydrase 1	ND	9	6	11	14
Protein 4.1	2	5	7	1	1
Parasite proteins					
HSP70 (PF3D7_0818900)	34	38	34	35	34
PV1	23	23	24	27	23
HSP101	ND	10	16	22	32
PTEX150	ND	2	9	7	4
RESA	2	15	15	8	14
MESA	2	17	41	11	24

Supplemental Table 3: Oligonucleotides used in this study. Restriction sites are underlined, InFusion overhangs are italicized, stop codons are bolded and mutations are in lower case. All oligonucleotides were purchased from Integrated DNA Technologies.

Oligonucleotide ID	Sequence
**PfEH1/2 into pPM2GT:**	
NJS1	*CACTATAGAACTCGAG*GATGGTGCTGAAAAGATATG
NJS2	*TGTGCTGCACCTGGCCTAGG*TGTGGTACATCCATTTTTATC
NJS4	*CACTATAGAACTCGAG*GAATGCCCTTATGAATACGG
NJS5	*TGTGCTGCACCTGGCCTAGG*TGTTTTATATTGACCATTTTTATC
**PfEH1/2 into pEOE and pTEOE:**	
NJS160	*CGATTTTTTCTCGAG*ATGAAAAAAGGAATGAAGAAATC
NJS161	*ATGTGCTGCACCTGGCCTAGG*TGTGGTACATCCATTTTTATC
NJS162	*CGATTTTTTCTCGAG*ATGAGAAATATTAATGGGTTC
NJS163	*ATGTGCTGCACCTGGCCTAGG*TGTTTTATATTGACCATTTTTATC
NJS299	*CGATTTTTTCTCGAG*ATGTCAGTTCACGAAAGGATACG
NJS300	*ATGTGCTGCACCTGGCCTAGG*TACAAATATATTATTTATCCAGTCAG
NJS301	*CGATTTTTTCTCGAG*ATGCTCAATTTTAGGAGGTGGAG
NJS302	*ATGTGCTGCACCTGGCCTAGG*TGGATATATATTATTAAGCC
**Mutagenesis of pTEOE_EH1:**	
NJS524	GAGGAAATTAAATTAAGATATTTAAGTGAAATTGAAAAAAATGATATATGTG
NJS525	GAGGAAATTAAATTAAGATATTTAAGTGAAGATGCATATGATGGTGCTG
NJS526	GAGGAAATTAAATTAAGATATTTAAGTGAAACACCAAAATTTTTACTGGATAG
NJS527	GAGGAAATTAAATTAAGATATTTAAGTGAACCTAGGCCAGGTGCAGCACAT
NJS528	GAATATAAGGAAAAATGTTACATTAAGTATGAACCTAGGCCAGGTGCAGCACAT
**PfEH1/2 into pET28a:**	
NJS49	*GTGCCGCGCGGCAGCCATATG*ACGGAATTAAATAAGAGGTC
NJS50	*GTGGTGGTGGTGCTCGAG***CTA**TGTGGTACATCCATTTTTATC
NJS55	*GTGCCGCGCGGCAGCCATATG*GTAATTAAAGATGGTATAGG
NJS56	*GTGGTGGTGGTGCTCGAG***CTA**TGTTTTATATTGACCATTTTTATC
NJS219	*GAAGGAGATATACCATGG*GAAGTGAAACGGAATTAAATAAGAGGTC
NJS220	*GTGGTGGTGGTGCTCGAG*TGTGGTACATCCATTTTTATC
NJS221	*GAAGGAGATATACCATGG*GATGTGATGTAATTAAAGATGGTATAGG
NJS222	*GTGGTGGTGGTGCTCGAG*TGTTTTATATTGACCATTTTTATC
**Mutagenesis of pET28a_EH1/2:**	
NJS124	GGAATTTTATTTGATTGGTGGTgCAATGGGTTGTTTAATAGCTGC
NJS 125	GCAGCTATTAAACAACCCATTGcACCACCAATCAAATAAAATTCC
NJS 128	CCTACTTTAATATTTTGTGGAGAAAAAGcTGATCTTTATGATGACGATG
NJS 129	CATCGTCATCATAAAGATCAgCTTTTTCTCCACAAAATATTAAAGTAGG
NJS 130	CATATTATTGTTTTCAAAGGAGAAAATgcTTATATAATACCTTCAAGAAC
NJS 131	GTTCTTGAAGGTATTATATAAgcATTTTCTCCTTTGAAAACAATAATATG
NJS134	GGAACATATGGAATAACAAATGCTGAACTTATTGGAAATAAAGACAAC
NJS 135	GTTGTCTTTATTTCCAATAAGTTCAGCATTTGTTATTCCATATGTTCC
NJS170	GGAATTTTATTTGATTGGTGGTgatATGGGTTGTTTAATAGCTGC

NJS171	GCAGCTATTAAACAACCCATatcACCACCAATCAAATAAAATTCC
**PfEH1/2 into pUF-TK:**	
NJS245	*CAGGCGCCAG<u>CCTAGG</u>*CATATGTTTTGGTTTGTATTG
NJS246	*ATCGATAACT<u>CCATGG</u>*CTTTATCTCCACCAAGCCCATG
NJS247	*AGATCTTCGG<u>ACTAGT</u>*GATTGGTGGTTCAATGGGTTG
NJS248	*CAATGGCCCCTTT<u>CCGCGG</u>*GTACATCCATTTTTATCAACAG
NJS249	*CAGGCGCCAG<u>CCTAGG</u>*CTTAAGTACATATGATAAAC
NJS250	*ATCGATAACT<u>CCATGG</u>*CTCAGATTCCGGATTCCCCTTC
NJS251	*AGATCTTCGG<u>ACTAGT</u>*CATTTGAACCTAGAAAATAAGG
NJS252	*CAATGGCCCCTTT<u>CCGCGG</u>*GTAAACATACATCATTGGGCG
**qRT-PCR:**	
EH1_primer1	TGGTGGTTCAATGGGTTGT
EH1_primer2	CCTAACATACCCACAGGTGATAAA
EH1_probe	5’ 6-FAM/AGCTGCTGC/ZEN/ATTTGCACAGAAAT/3’ IABkFQ
EH2_primer1	CACAATCCATACTTGGTTGCATAA
EH2_primer2	CCTCTGAACAATATTCATCATCCTTTC
EH2_probe	5’ 6-FAM/TTTGCATAT/ZEN/GTGGAGTGCTCATCA/3’ IABkFQ
Actin_primer1	TGTTGACAACGGATCAGGTAAT
Actin_primer2	CCTGGATTCTTTGGTCTTCCTAC
Actin_probe	5’ 6-FAM/AGGAGTTGC/ZEN/AGGAGATGATGCACC/3’ IABkFQ

\
